# Glycemic load impacts the response of acquired resistance in breast cancer cells to chemotherapeutic drugs in vitro

**DOI:** 10.1371/journal.pone.0311345

**Published:** 2024-11-22

**Authors:** Sirin A. Adham, Azza Al Kalbani, Noura Al Zeheimi, Muna Al Dalali, Noor Al Kharusi, Azeeza Siddiqi, Aliya Al Maskari

**Affiliations:** Department of Biology, College of Science, Sultan Qaboos University, Muscat, Oman; Brigham and Women’s Hospital, UNITED STATES OF AMERICA

## Abstract

Resisting chemotherapy is a significant hurdle in treating breast cancer. Locally advanced breast cancer patients undergo four cycles of Adriamycin and Cyclophosphamide, followed by four cycles of Paclitaxel before surgery. Some patients resist this regimen, and their cancer recurred. Our study aimed to understand the underlying mechanisms of acquired resistance during these specific treatment phases. We explored how breast cancer cells, resistant to chemotherapy, respond to different glucose levels, shedding light on the intricate relationship between diabetes, breast cancer subtype, and resistance to preoperative chemotherapy. We examined two groups of cell lines: the standard MDA-MB-231 and MCF7 cells and their resistant counterparts after exposure to four cycles of Adriamycin and cyclophosphamide (4xAC) or four cycles of 4xAC and Paclitaxel (4xAC+4xPAC), aiming to unravel the mechanisms and cellular responses at these critical treatment stages. Notably, under normal and low glucose conditions, the resistant MDA-MB-231 cells showed accelerated growth compared to the control cells, while the resistant MCF7 cells proliferated more slowly than their original counterparts. Resistance to 4xAC resulted in significant cell death in both cell lines, especially under low glucose conditions, in contrast to control or 4xAC+4xPAC-resistant cells. The similarity between the MCF7 4xAC+4xPAC resistant cells and the control might be due to the P-AKT expression pattern in response to glucose levels since the levels were constant in MCF7 4xAC in all glucose concentrations. Molecular analysis revealed specific protein accumulations explaining the heightened proliferation and invasion in resistant MDA-MB-231 cells and their ability to withstand low glucose levels compared to MCF7. In conclusion, increased drug involvement corresponds to increased cell resistance, and changes in glucose levels differentially impact resistant variant cells to different drugs. The findings can be translated clinically to explain patients’ differential responses to preoperative chemotherapy cycles considering their breast cancer subtype and diabetic status.

## Introduction

Breast cancer (B.C.) is now the most diagnosed cancer in females, with around 2.3 million new cases in the last couple of years [1]. Multidrug resistance, once acquired, typically results in cancer recurrence. In the tumor microenvironment, elevated glucose levels are crucial in fueling tumor growth and advancing disease progression [2]. Diabetes is a risk factor for many cancers, including breast cancer [3]. The mammary adipocytes and mesenchymal stem cells (MSCs) produce a variety of extracellular matrix components, growth factors, and cytokines that support breast cancer progression [4]. Studies indicated that the glucose-lowering drug metformin acts best against triple-negative breast cancer compared with luminal BC [5]. Other studies indicated that diabetic patients harbouring HER2-positive tumors benefit from metformin [6]; however, metformin in cancer treatment is still under investigation [7]. In this study, we aimed to combine the triangle of neoadjuvant chemotherapy resistance, glycemic index, and breast cancer subtype to understand the correlation among the three factors on the progression of breast cancer cells. The two main manifestation of diabetes are the hyper and hypo glycemia both are correlated to produce oxidative stress on the cells [8] which also play a role in genomic instability and cancer [9, 10]. Studies are emphasizing on cancer diagnostics in diabetic patients as protocol for primary prevention and early detection [11]. A recent study indicated that diabetes is not associated with increased mortality of patients over five years with metastatic breast cancer; however, it is associated with the worst overall survival among a cohort of longer-term survivors [12]. In another study, the increase in .C.BC mortality is positively correlated with type 2 diabetes mellitus when diagnosed before BC [13]. Anti-metabolic drugs along with chemotherapy showed synergistic effects in triple-negative breast cancer cells MDA-MB-231, MDA-MB-468, and HCC1143 [14]. One of the mechanisms by which cancer cells become resistant to drugs is the disruption of metabolic activity in solid tumors that can be mainly caused by transient hypoxia that is associated with glucose deprivation, which leads to disrupted protein folding in the endoplasmic reticulum conferring resistance to topoisomerase II–targeted drugs [15].

High blood sugar levels, known as hyperglycemia, pose a substantial risk factor for the progression of breast cancer [16]. Studies have demonstrated that hyperglycemia is associated with increased prevalence and mortality rates in breast cancer. Additionally, hyperglycemia has been shown to influence the development of chemoresistance in this context [17]. Interestingly, glucose deprivation or hypoglycemia resulted in more resistant phenotype on breast cancer cells resistant to the EGFR inhibitor lapatinib and overcoming the networks responding to glucose deprivation show promise in overcoming the resistant phenotype and diminishing the survival of resistant cells [18].

The insulin growth factor receptor 1 (IGF-1R) with the proliferation marker Ki67 are shown to act as prognosis markers associated with patients harboring diabetes mellitus and breast cancer [19]. Acquired chemotherapy resistance in locally advanced .C.BC can happen due to different molecular changes by which the cells acquire the expression of certain proteins such as p-glycoprotein [20]. Tumor cells microenvironment is complex network of signaling and it is believed to play a role in acquired resistance to chemotherapy [21]. Locally advanced breast cancer patients receive Adriamycin and cyclophosphamide followed by Paclitaxel cycles [22]. To our knowledge, no studies have explored acquired resistance during neoadjuvant treatment time points and the impact of the glycemic load. Therefore, we identified the cellular and molecular alterations in both control MDA-MB-231 and MCF7 cells, as well as their resistant variants to Adriamycin plus cyclophosphamide (A.C.). We also investigated the resistant variant to these drugs combined with Paclitaxel (AC+PAC) (generated in our prior research [23]); the established resistant MCF7 cells are HER2 positive, and their response to the different glycemic conditions showed a distinct response which can be translated to the patients that are diagnosed with one subtype at their initial screening and their subtype changes after they are treated with neoadjuvant chemotherapy.

## Results

### Breast cancer cells proliferation varies with glycemic levels and cancer subtype

**Fig 1:**
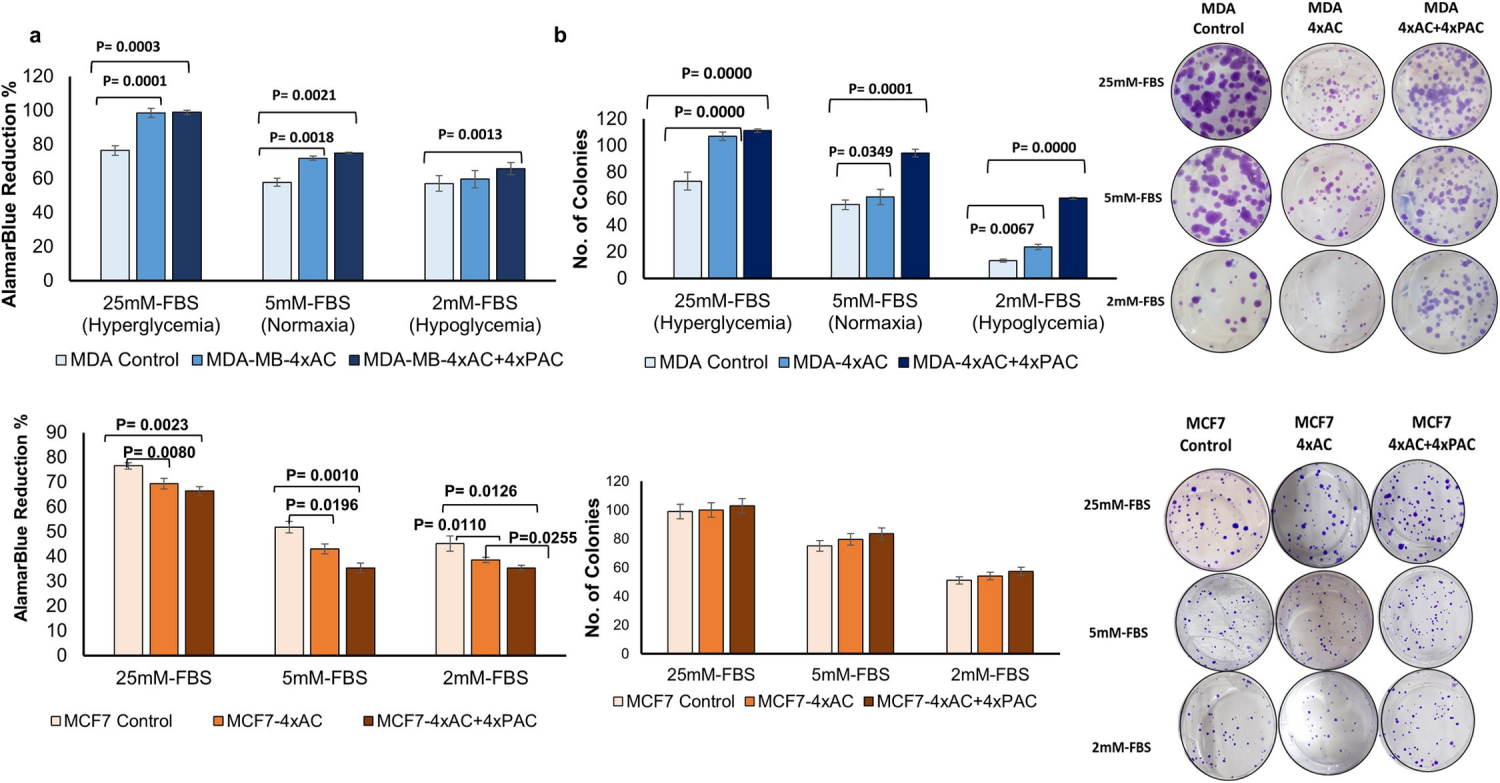
Glucose levels govern cell proliferation. (a&b Top panel) Resistant MDA-4xAC and MDA-4xAC+4xPAC proliferated and produced more colonies compared to control cells under hyperglycemia (25 mM) and normal glucose level (5 mM). Resistant MCF7-4xAC and MCF7-4xAC+4xPAC proliferated and had fewer and smaller colonies than control MCF7 cells (bottom panel).

**Fig 2:**
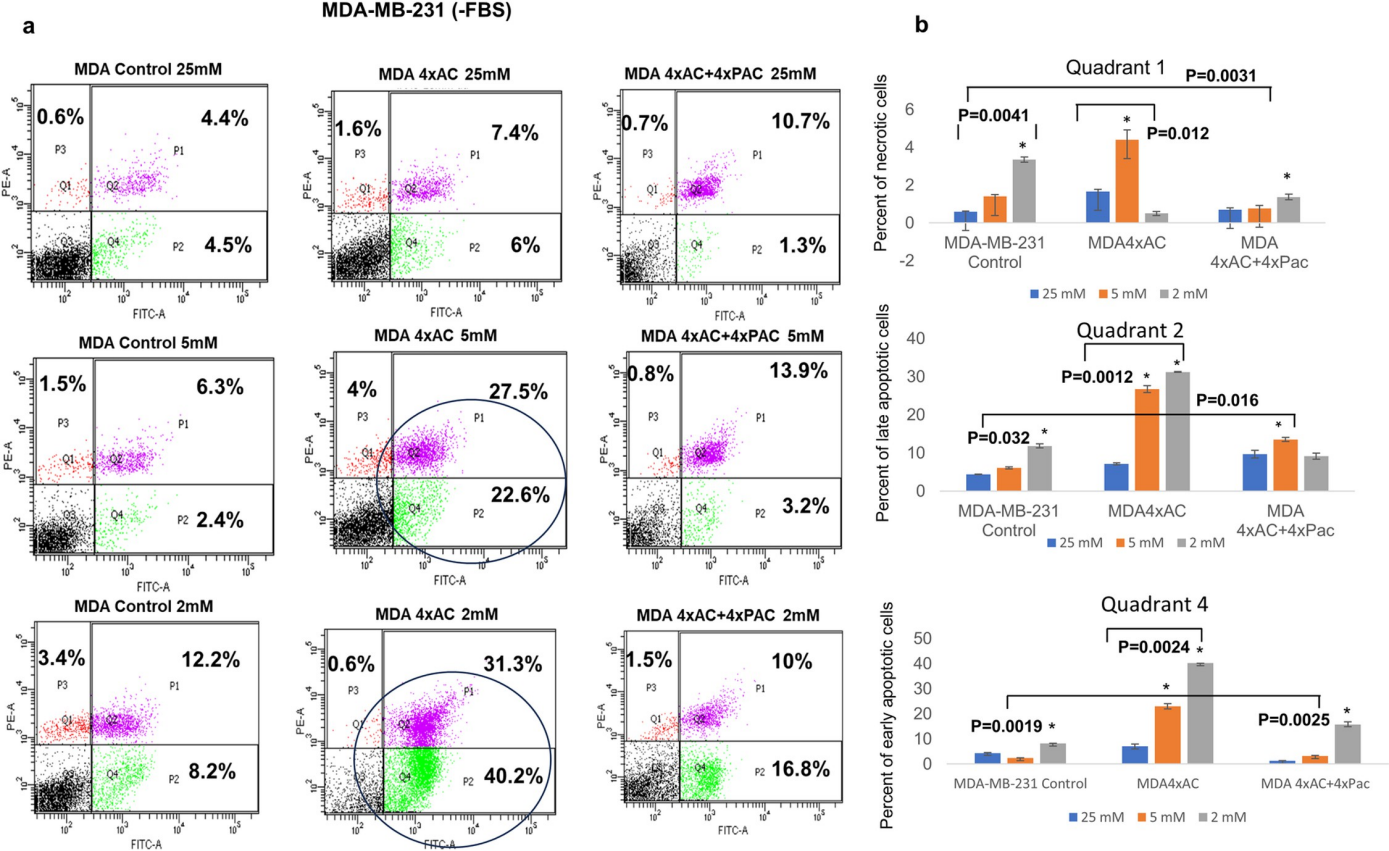
MDA 4xAC highly glucose sensitive. (a) Representative flow cytometry plots of MDA-MB-231 control and resistant variant cells under 25 mM, 5 mM, and 2 mM glucose levels. Numbers in quadrants show the average percentages of necrotic (.I.PI stained), early (FITC stained), and late apoptotic cells population (double stained PI&FITC). (b) Graphs summarizes the results of three independent flow cytometry experiments quantifing the mean ±SD of MDA control, MDA 4xAC, and MDA 4xAC+4xPac of necrotic cells in quadrants #1 late apoptotic cells in quadrants #2, and early apoptotic cells in quadrants #4. * represents p value less than 0.05.

All cells showed significant decrease in the proliferation ability when exposed to normal glucose level and hypoglycemia for 24 hours when compared with the usual used DMEM media containing hyperglycemic concentration of glucose (25 mM) (Fig 1A). Interestingly, under all three different glycemic conditions, MDA-MB-231 resistant cells to 4xAC and to 4xAC+4xPAC significantly proliferated faster than the control cells (1A). In addition, under hypoglycemia the resistant MDA-MB-231 variants to 4xAC+4xPAC showed a significant increase in proliferation in comparison to the resistant variant to 4xAC or control cells (Fig 1A). Unlike MDA-MB-231 cells, resistant MCF7 proliferated significantly slower than the control MCF7 cells (Fig 2B). In addition, under hypoglycemia the resistant variants to 4xAC+4xPAC showed a significant decrease in proliferation in comparison to the resistant 4xAC variant. The results represent the mean value ± .D.SD of three independent experiments.

### Resistant MDA-MB-231 cells showed increased clonogenicity with rising resistance and glucose, unlike MCF7 resistant cells

Consistent with the Warburg effect, hypoglycemia significantly decreased the number of colonies in all control and resistant cells (Fig 1B).

Under hyperglycemia (25 mM) the resistant MDA-MB-231 cells to 4xAC and 4xAC+4xPAC produced significantly higher numbers of colonies compared with control cells. Under normal 5 mM and hypoglycemia 2mM the resistant cells to MDA-4xAC+4xPAC significantly produced more colonies than resistant MDA-4xAC or control cells. Conversely, MCF7 resistant cells did not show any significant increase in the number of colonies compared with the control cells under the three glucose levels and the size of the colonies was smaller in both resistant variant cells (2, 5 and 25 mM) (Fig 1B).

### Adriamycin and cyclophosphamide (4xAC) resistance in MDA-MB-231 and MCF7 cells sensitized the cells to glucose deprivation

The cells grown in media without FBS, and three glycemic conditions for 24 hours were subjected to flow cytometry annexin V analysis. The results revealed that MDA-4xAC cells are highly sensitive to low glucose levels (2 mM), showing a total of 71.5% early and late apoptotic cells and a minimal necrosis 0.6%. In comparison, the control MDA-MB-231 and MDA-4xAC+4xPAC cells exhibited lower levels of early and late apoptotic cells, 20.4% and 26.8% respectively (Fig 2A and 2B). Even under normal glucose levels (5 mM), MDA-4xAC cells still had the highest percentage of early and late apoptotic cells a total of 50.1%. However, in high glucose conditions (25 mM), the differences between the resistant variants and control MDA-MB-231 cells were not significant (Fig 2A and 2B). The astaristic * represents a significant difference between different glucose levels (25 mM, 5 mM, 2 mM) of the same cell type mean ±SD of the percentage of cells’ death events ≤0.05.

**Fig 3:**
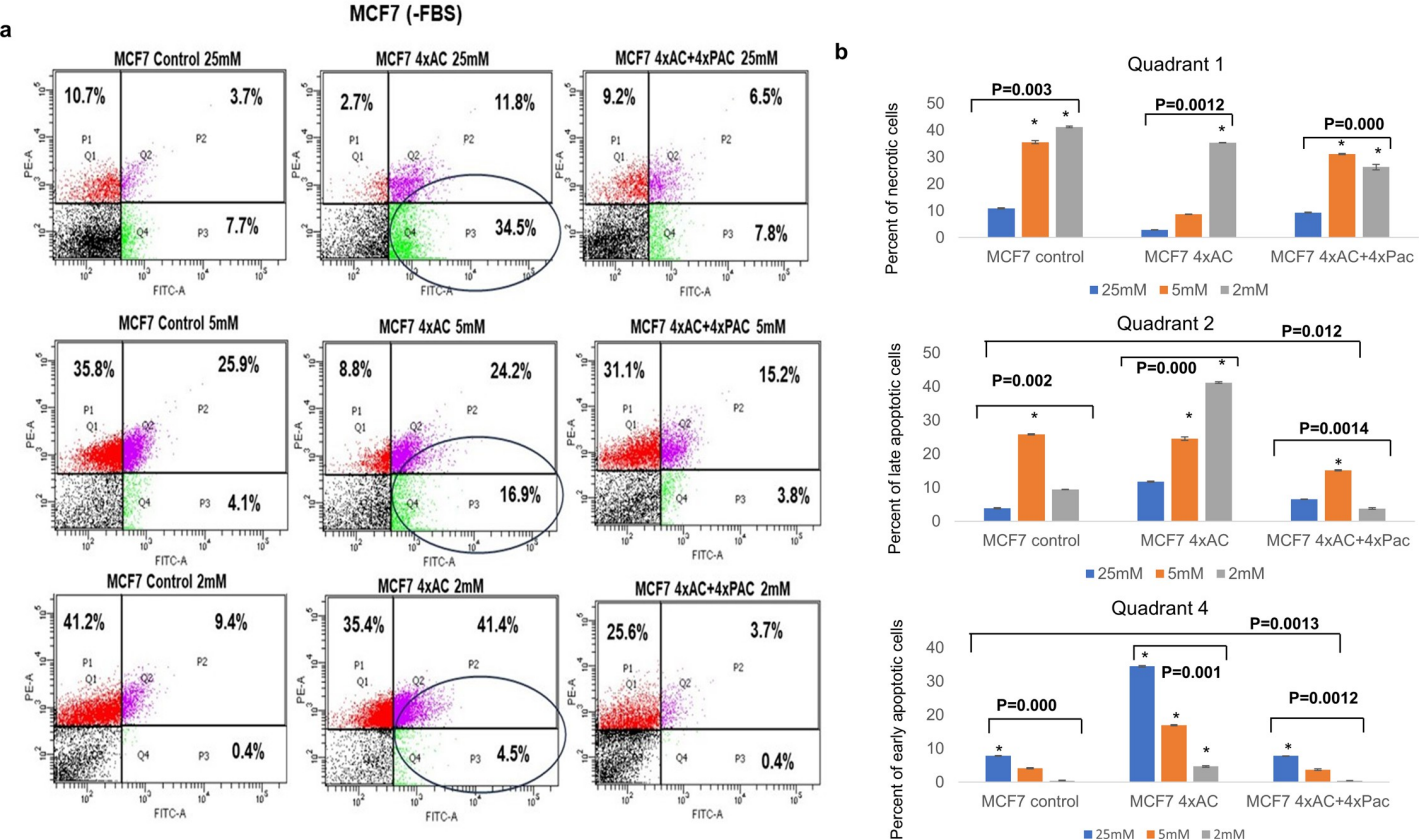
MCF7 4xAC highly glucose-sensitive. (a) Representative flow cytometry plots of MCF7 control and resistant variant cells under 25 mM, 5 mM, and 2 mM glucose levels. Numbers in quadrants show the mean percentages of necrotic (PI stained), early (FITC stained), and late apoptotic cells population (double stained PI&FITC). (b) Graphs summarizes the results of three independent flow cytometry experiments quantifying the mean ±SD of MDA control, MDA 4xAC, and MDA 4xAC+4xPac of necrotic cells in quadrants #1 late apoptotic cells in quadrants #2, and early apoptotic cells in quadrants #4. * represents *p* value less than 0.05.

Similarly, MCF7-4xAC cells demonstrated high sensitivity to glucose deprivation (2 mM) with 45.9% early and late apoptotic cells and 35.4% necrotic cells. In contrast, both MCF7 control and the variants resistant to MCF-4xAC+4xPAC cells had low percentages of early and late apoptotic cells (9.8% and 4.1% respectively) but a high percentage of necrosis under low glucose conditions (2 mM) (Fig 3A and 3B). Notably, under normal (5 mM) and high glucose levels (25 mM), MCF7-4xAC still exhibited higher percentages of early and late apoptotic cells (41.2% and 46.3% respectively) with a lower percentage of necrotic cells. Conversely, both control MCF7 and MCF7-4xAC+4xPAC had lower percentages of early and late apoptotic cells under normal (5 mM) (30% and 19% respectively) but high percentages of necrosis (35.8% and 31.1%). Under high glucose conditions (25 mM) MCF7-4xAC had the highest percentage of early and late apoptotic cells (46.3%) when compared with control and MCF7-4xAC+4xPAC (Fig 3A and 3B). The astaristic * represents a significant difference between different glucose levels (25 mM, 5 mM, 2 mM) of the same cell type ±SD of the percentage of cells’ death events ≤0.05.

### Hyperglycemic conditions protected the chemo-resistant cells to 4xAC+4xPAC from the action of Paclitaxel treatment for 24 hours

Control and resistant cells underwent an additional 24-hour Paclitaxel treatment under three glucose concentrations without FBS. Under hypoglycemia (2 mM), MDA-4xAC exhibited the highest apoptotic events (48.9%), control MDA-MB-231 cells showed (37%), and MDA-4xAC+PAC had the lowest (22.4%). Higher glucose levels correlated with decreased early/late apoptosis (S1A Fig). In MCF7, Paclitaxel’s impact on control and resistant cells was more pronounced, especially given MCF7’s non-invasive nature [24]. In line with the abovementioned findings, MCF7-4xAC exhibited the highest susceptibility under hypoglycemia (2 mM glucose), with 52.2% necrotic cells. Hypoglycemia reduced the early apoptotic cell population in all control and resistant MCF7 variants. Subsequently, early apoptotic cells increased under normal glucose levels while necrosis decreased in control MCF7 and resistant MCF7-4xAC but not in MCF7-4xAC+4xPAC. Hyperglycemia (25 mM) led to an increase in early apoptotic cells in control MCF7 (34.6%), resistant MCF7-4xAC (14.7%), and, to a lesser extent, in MCF7-4xAC+4xPAC (3.8%), accompanied by a significant reduction in overall cell death events (45.8% in control MCF7, 27.1% in MCF7-4xAC, and 19.1% in MCF7-4xAC+4xPAC) (S1A and S1B Fig).

### MDA-MB-231 and MCF7 control and resistant cells invaded differently under different glycemic conditions

**Fig 4:**
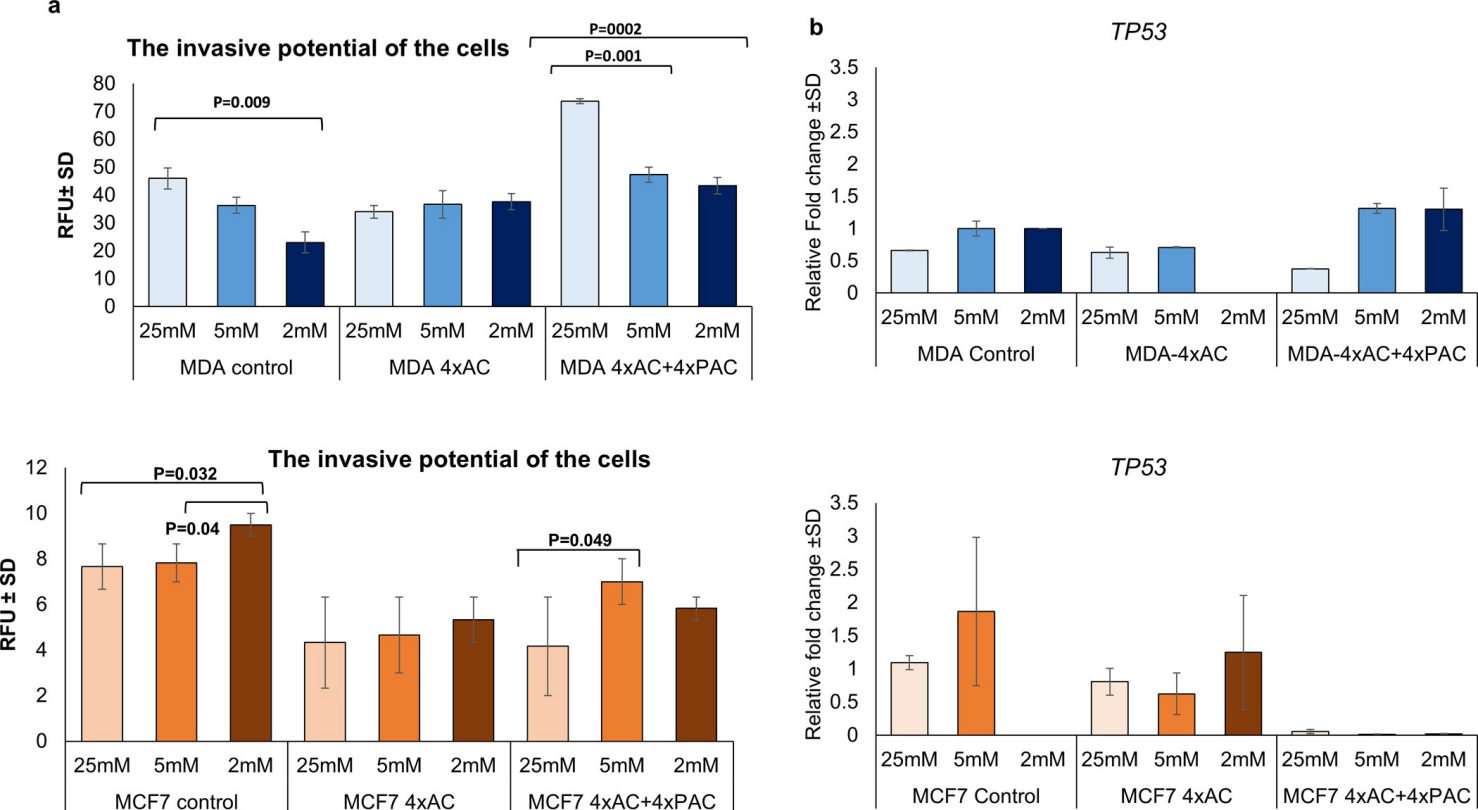
Constant invasion in 4xAC resistance. **(a)** The Top graph displays MDA-MB-231 control and resistant cell invasion under three glucose levels (25 mM, 5 mM, and 2 mM). The bottom graph illustrates MCF7 control and resistant cell invasion. **(b)** Real-time PCR results for TP53 transcripts in MDA-MB-231 and MCF7 control and resistant cells under 25 mM, 5 mM, and 2 mM glucose. The experiments were replicated three times independently; significance was considered at P<0.05.

The invasion of MDA-MB-231 control cells increased with increasing glucose concentration however, in MDA-4xAC there was no significant change in invasion among the three glucose levels. In MDA 4xAC+4xPAC there was no significant increase between the hypoglycemia (2mM) and normal glucose level (5mM) (Fig 4A) but the increase in invasion was significantly higher under hyperglycemia (25mM). In MCF7 control cells, the invasion decreased by decreasing the glucose to normal (5mM) and hypoglycemic (2mM) levels. In MCF7-4xAC there is no change in the invasion ability under different glucose levels. In MCF7-4xAC+4xPAC, the invasion ability of the cells was increased under normal glucose level (5mM) when compared with hyperglycemia (25mM) (Fig 4A).

### The apoptotic gene *TP53* was significantly down regulated under all glucose levels in MCF7-4xAC+Pac but not MCF-7 4xAC

In MDA-MB-231 control cells, *Tp53* levels were low but decreased under hyperglycemia (25 mM). In resistant MDA-4xAC, Tp53 was undetected under hypoglycemia, increased at normal (5mM) and hyperglycemic (25mM). MDA-4xAC+4xPAC showed Tp53 expression like control cells, decreasing at hyperglycemia (25mM) (Fig 4B). In MCF7 control cells, the *TP53* levels showed a trend of increase under normal glucose levels (5mM). In resistant MCF7-4xAC the levels of *TP53* were reduced under normal (5mM) and hyperglycemia (25 mM). However, in resistant MCF7-4xAC+4xPAC significantly reduced the *TP53* levels under all glycemic conditions (Fig 4B).

### Western blot analysis reveals that NRP1 protein is expressing constantly in resistant MDA and MCF7-4xAC+4xPAC regardless of glucose level in the microenvironment

**Fig 5:**
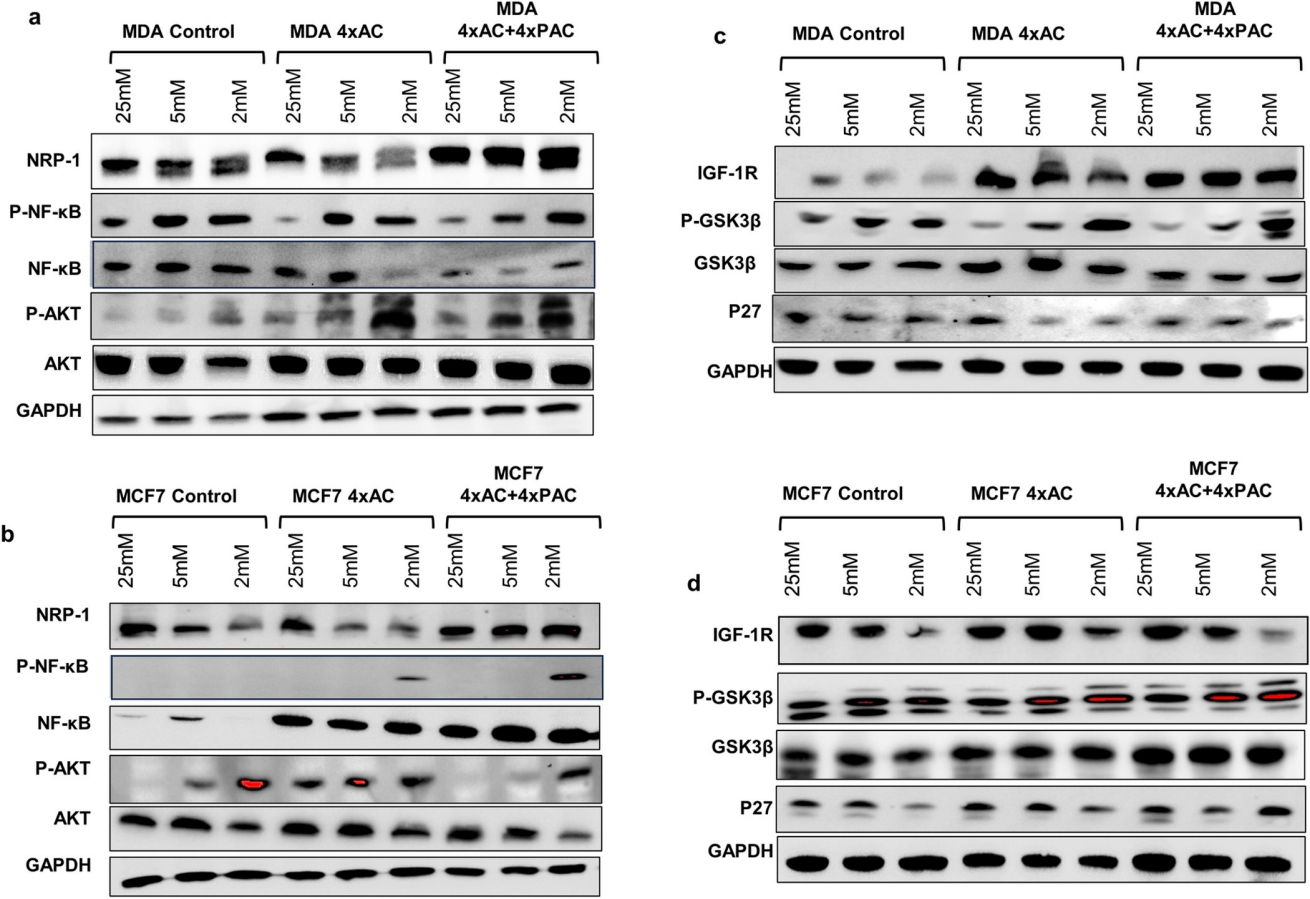
Signaling pathway analysis using immunoblots. Panel (**a)**, western blot analysis shows the blots of NRP-1, P-NF-kB, NF-kB, P-AKT-473, AKT, and GAPDH of MDA-MB-231 control and resistant variants under 25 mM, 5 mM, and 2 mM of glucose. Panel (**b)** shows the same proteins but in MCF7 control and resistant variants. Panel (**c)**, western blot analysis shows the blots of IGF1R,P-GSK3β, GSK3β, P27 and GAPDH of MDA-MB-231 control and resistant variants under 25 mM, 5 mM, and 2 mM of glucose. Panel (**d)** shows the same proteins but in MCF7 control and resistant variants. The graphs of the figures show the protein bands’ mean relative intensity.

In both resistant cell lines MDA-4xAC+4xPAC, and MCF7-4xAC+4xPAC it is well noticed that the NRP1 protein is expressed with almost equal quantities in the three-glycemic conditions used with a slight increase at 2 mM. On the other hand, in control and resistant cells MDA- 4xAC and more obviously in MCF7-4xAC and, the expression of NRP1 protein decreases proportionally with reducing the glucose concentration (Fig 5A and 5B).

### Expression of P-AKT and P-NF-kB has a distinct expression pattern between MCF7 and MDA-MB-231 control and resistant variants

The AKT and NF-kB survival signaling pathways were upregulated under hypoglycemic conditions. Interestingly, the p-AKT levels in control MDA-MB-231 cells were increased under hypoglycemia and the levels in both resistant variants were higher than the control cells (Fig 5A). Generally, the NF-kB levels were decreased at hypoglycemic conditions (2 mM) notably in resistant cells to MDA-4xAC. However, the levels of NF-kB in MDA-4xAC+4xPAC were reduced at 5 mM. P-NF-kB levels in control MDA-MB-231 cells were increased in both resistant variants by decreasing glucose concentration (Fig 5A).

In the control and resistant MCF7 variant cells the p-AKT levels were increased under hypoglycemia. Notably, in MCF7-4xAC, p-AKT was upregulated in the three glycemic conditions. The survival molecule NF-kB was down regulated in the parental MCF7 cells however its levels were consistently expressed in the resistant variants under all glucose concentrations. The phosphorylated form of NF-kB was exclusively increased under hypoglycemia in the resistant variants of MCF7 cells (Fig 5B).

### In hypoglycemia, IGF1R receptor remained unchanged in MDA-4xAC+4xPAC across all glucose levels, whereas it decreased in control and resistant MCF7 cellsC

In control MDA-MB-231 and resistant MDA-4xAC cells, IGFR1 decreased with lower glucose. Surprisingly, IGFR1 levels in resistant MDA-4xAC+4xPAC cells remained unaffected by glucose changes (Fig 5C). In control and resistant MDA-MB-231 cells, P-GSK3β levels increased with normal (5 mM) and hypoglycemic (2 mM) conditions (Fig 5C).

In control and resistant MCF7 cells the IGFR1 was profoundly decreased under the hypoglycemic condition. In MCF7 control and resistant MCF7-4xAC and MCF7-4xAC+4xPAC the P-GSK3β levels remained constant under all glycemic conditions (Fig 5D).

### The cell cycle protein P27 expression had distinct pattern between control and resistant MBA-MB-231 and MCF7

There was a slight decrease in the cell cycle protein p27 in response to the decrease in glucose levels in normal and hypoglycemia (5mM & 2mM) in resistant MDA-4xAC and only under hypoglycemia (2mM) in MDA-4xAC+4xPAC. However, there was no change observed in control MDA-MB-231 cells (Fig 5D). In MCF7 cells, the control and MCF7-4xAC showed a decrease in P27 under hypoglycemia (2mM). However, the MCF7-4xAC+4xPAC exhibited a decrease in P27 under normal glucose levels (5mM) (Fig 5D).

## Discussion

Acquired multidrug resistance is an obstacle that usually leads to the recurrence of cancer [25]. Tumor core tissue is known to be hypoxic and/or hypoglycemic, which was shown to play a major role in reducing the efficacy of anticancer drugs [26, 27]. Therefore, depriving cells of glucose could potentially contribute to the emergence of oncogenic mutations that promote cell growth during tumor development [28]. In this study, we used two breast cancer cell lines in which we investigated the effect of glycemic load on the control versus their resistant variants to neoadjuvant chemotherapy. Consistent with the Warburg effect, all cells exhibited a notable reduction in proliferation after 24 hours of exposure to both normal glucose levels and hypoglycemia, compared to the standard DMEM media with hyperglycemic glucose concentrations 4.5 g/L (25 mM). However, comparing the effect on the control and resistant cells at the three glucose concentrations, the glycemic load has a distinctive effect between the two cell lines used on the cellular and molecular levels.

Collectively, the different experiments done in this research supported the fact that the resistant MCF 7 cells are more sensitive to glycemic change compared with resistant MDA-MB-231 cells, which is supported by the background knowledge of the two cell lines MCF7 is less aggressive and will be affected more than the aggressive MDA-MB-231 cells [24]. Since neoadjuvant chemotherapy is given sequentially, Adriamycin cyclophosphamide (A.C.) followed by Paclitaxel [29], the resistant cells to the initial treatment with .C.AC only are more sensitive to glucose deprivation and showed a significant increase in the cell death events when compared with the cells resistant to all three drugs 4xAC+4xPAC, supporting the fact that the more aggressive the treatment is the more resistance it becomes. Although adding Paclitaxel to the treatment regimen increased survival [30], not all patients responded equally; some developed resistance after the complete AC+ PAC treatment, which might suggest that the remaining resistant cells are much more aggressive than those that stay if the patient was only treated with .C.AC only which need further confirmation on a clinical level.

The annexin V assay supported the findings of the proliferation and clonogenicity assays in that hypoglycemia caused increased cell death events in MCF7 control & resistant cells with a high percentage of necrotic cell population compared to control and resistant MDA-MB-231 cells that the percentage of necrotic population is negligible. The additional cycle of Paclitaxel treatment in control and resistant variant cells provided supportive evidence that glucose protected the cells and reduced the cell death events proportionally.

When examining the y-axis of the graphs in Fig 4A, it is evident that MDA-MB-231 cells invade much more than MCF7 cells. However, the invasion of both resistant variants, MCF7-4xAC and MDA-4xAC, did not change across the three glucose levels. This consistency aligns with their high sensitivity to glucose deprivation. Conversely, the resistant MDA-4xAC+4xPAC exhibited a significant increase in invasion ability with higher glucose availability (5 mM and 25 mM). In contrast, the invasion of resistant MCF7-4xAC+4xPAC showed a trend of increase at normal glucose levels (5mM). Notably, control MDA-MB-231 cells invaded less as the glucose concentration decreased; however, control MCF7 invasion increased with decreasing glucose, which can, in part, support the fact that tumor microenvironment affects the process of metastasis based on the subtype of BC [31, 32]. From the above results, we can see that the response of breast cancer cells to glucose levels and treatment depends primarily on the subtype of breast cancer and the drugs received, which suggests considering diabetes and subtypes of breast cancer for the treatment decision.

Since *TP53* mutation influences energy metabolism and plays a major role in the cell cycle and apoptosis [33, 34], we quantified the expression levels of *Tp53* at the different glycemic concentrations. However, the detected transcript levels were very low; the levels reduced notably in response to Paclitaxel resistance at all glucose concentrations in MCF7-4xAC+4xPAC and a less extent in MCF7-4xAC cells. Interestingly, *Tp53* showed a trend of increase in MCF7 4xAC at 2 mM concentration, explaining the highest number of cell death.

Molecularly, the protein Neuropilin-1 expression is correlated with chemotherapy resistance in MDA-MB-231 and MCF7 cells [23, 35] besides our recent finding about its involvement in lung metastasis in NRP-1 knockout MDA-MB-231 cells [36]. In this study, NRP-1 was upregulated in resistant MDA-MB-231 and MCF7 cells to 4xAC+4xPAC regardless of the glucose concentration; however, it was affected by the decrease in glucose in the control and resistant cells to 4xAC, the fact that supports its involvement in the most resistant cells to all drugs used .C.AC with Pac.

Previously, we showed that the resistant MCF7-4xAC and MCF7 4xAC+4xPAC over-expressed HER2 [23]. It is well known that HER2-positive breast cancer cells regulate metabolism through the *PI3K/AKT* pathway [37, 38]. Akt activation and upregulation in MCF7-4xAC were distinct from the control MCF7 and MCF7-4xAC+4xPAC. In MCF7-4xAC, Akt activation (phosphorylation) was equal under all glucose concentrations, while in control MCF-7 and MCF7-4xAC+4xPAC activation only increased under normal and more obvious under hyperglycemia (25 mM) (Fig 5). The distinct pattern of Akt in MCF7-4xAC might be in part due to the .C.AC treatment since the MCF-7 4xAC+4xPAC had the same pattern of Akt as the control cells, which implies that the additional cycles of Paclitaxel restored the pattern after the .C.AC resistance this results is consistent with our previous finding as AC resistant cells had a distinct protein expression pattern to those in control untreated or resistant MCF7 to 4xAC+4xPAC [23]. Since the cells were trying to survive under low glucose levels, P-AKT expression was constant under all glucose levels only in MCF7-4xAC, confirming their sensitivity observed in Annexin V results. Although the MDA-4xAC was also the most sensitive when compared with control MDA-MB-231 and MDA-4xAC+4xPAC, the Akt phosphorylation increased as glucose decreased, supporting survival and higher proliferation ability when compared with MCF7. Therefore, Convergence and divergence between MCF7-4xAC and MDA-4xAC might be in part due to the HER2 status; in other words, the subtype of the cells since resistant MDA-MB-231 cells remained HER2 negative and therefore, the pattern of Akt seen in MCF7-4xAC is different from the pattern seen in MDA-4xAC. This is a notion that can be translated to explain previous clinical studies that showed not every diabetic patient with breast cancer can benefit from metformin treatment, only those who have HER2 positive tumors since HER2 is correlated with metabolism through the activation of the downstream signaling of Akt [6].

In MDA-MB-231 control, MDA4xAC, and MDA-4xAC+4xPAC, the levels of P-NF-kB increased as the glucose decreased, supporting the survival of the cells. In MCF7 control, the phosphorylated form of NF-kB was not detected and exclusively increased under hypoglycemia (2 mM) in the resistant variants of MCF7-4xAC and MCF7-4xAC+4xPAC cells (Fig 5), consistent with the fact that the higher expression of p-NF-kB is correlated with more resistant cells to chemotherapy [39].

The decrease in phosphorylated GSK3β is responsive to glucose levels [40]. It is known that Akt phosphorylates GSK-3β on S9, resulting in its inactivation [41]. The phosphorylated levels of P-GSK3β were increased under normal glucose levels (5 mM) and hypoglycemia (2mM) in control MDA-MB-231 and MCF7, indicating its inactivation. The profound increase in P-GSK3 β was more detected in resistant variants of both cell lines (Fig 5B), which is in line with a previous study that showed blocking GSK-3β activity may lead to drug and hormonal resistance and modify sensitivity to targeted therapy in MCF-7 breast cancer cells [41]. Ligand binding to IGF-1R activates the phosphatidylinositol 3-kinase (PI3K)/Akt/mammalian target of rapamycin (mTOR) [42]. MDA-MB-231 control cells had lower IGF-1R protein expression, while resistant cells showed higher levels. The MDA-4xAC+4xPAC consistently upregulated IGF1R under various glucose conditions, indicating its constant requirement for cell resistance. Interestingly, IGF1R significantly decreased under hypoglycemic conditions (2 mM), especially in MCF7-resistant cells (Fig 5A and 5B). This reduction in IGF1-R levels may explain the reduced proliferation and invasion ability of MCF7 cells, aligning with previous reports linking increased IGF-1R activity to cancer cell proliferation, migration, and invasion [43, 44]. An earlier study showed that glucose starvation induces proteasome-dependent degradation of cytoplasmic p27, accompanied by a decrease in cell motility [42]. Another study showed that the metformin-resistant cell line MDA-MB-231 expresses significantly lower levels of p27 than the metformin-sensitive cell line MCF7 [45]. In MDA-4xAC, P27 protein was downregulated under normal and hypoglycemic conditions, contrasting with control MDA-MB-231 and resistant MDA-4xAC+4xPAC, where decreased glucose had no impact on P27 protein levels. This aligns with Annexin V results, highlighting MDA-4xAC’s heightened sensitivity to glucose starvation and additional paclitaxel treatment (Figs 2A and 3A). In MCF7, P27 downregulation occurred only under hypoglycemia in MCF7 control and MCF7-4xAC but not in MCF7-4xAC+4xPAC; this further supports flow cytometry findings that Paclitaxel-treated cells exhibited the lowest percentage of necrotic cells.

Overall, our findings highlight that breast cancer subtype, glucose levels, and the specific drugs used influence the response to treatment.

## Materials and methods

### Study design, cell lines, and cell culture

MCF7 (300273) and MDA-MB-231 (300275) were purchased directly from Cell Lines Service CLS (Germany), which authenticates cell lines using the STR DNA analysis method. The parental MCF7 and MDA-MB-231 cells were used along with two resistant variants of MDA-MB-231 (4xAC and 4xAC+4xPAC) and two resistant variants of MCF7 (4xAC and 4xAC+4xPAC) in this study. We generated the resistant variant cells in a previous research project, the cells were authenticated in 2021 and were checked for mycoplasma infection [23]. The MDA-MB-231 control and resistant cells were grown and cultured in Dulbecco’s Modified Eagle medium (DMEM) (4500 mg glucose/L) (SIGMA Aldrich, Germany) however, MCF7 cells grew in Opti-MEM media (Gibco, Germany) containing both culture media were supplemented with 10% Fetal Bovine Serum (FBS) (Gibco ^TM^), 1% (v/v) sodium pyruvate (Sigma-Aldrich) and 0.5% (v/v) of gentamicin (Invitrogen ^TM^). All cells were grown and maintained at 37° C in a humidified incubator containing 5% CO2.

### Growing under different glucose concentrations

When the confluency of the cell lines reached 80% in 10 cm plate, they were washed with PBS and the normal growth media was replaced with 10 ml of DMEM containing different glucose concentrations. Those concentrations were 25 mM, 5mM and 2mM (Sigma, Germany); these three concentrations represented hyperglycemia, normal and hypoglycemia conditions respectively.

### Western blot

After glucose exposure, the culture medium was removed, and cells were washed with ice-cold PBS. Cells were then lysed using 1X Cell Signaling Technology’s buffer with protease inhibitor PMSF (Merck), and the resulting lysate was collected after centrifugation. A 100 μg of protein lysate was loaded onto a polyacrylamide gel. The protein samples were mixed with a sample buffer, heated, and underwent SDS-PAGE using a MiniPROTEIN Tetra Cell (Bio-Rad, USA). After gel electrophoresis, proteins were transferred to a PVDF membrane. After a blocking step in 5% milk, the membrane was incubated overnight with 1:1000 dilution of all primary antibodies used (S1 Table). After multiple washes, the membrane was incubated with a peroxidase-conjugated secondary antibody, and signal detection was achieved using an ECL detection kit (Bio-Rad, USA). Signal detection was achieved using an ECL detection kit (Bio-rad, USA), and the expression picture was developed and analyzed for band intensity using the Chemidoc touch system and Image Lab software (Bio-rad, USA).

### Proliferation assay

Cell viability was assessed using the Alamar Blue Assay (Invitrogen, Carlsbad, CA). Briefly, 8×10^4 cells were seeded per well in a 96-well plate (Corning Incorporated, USA) with 200μl complete growth medium. After 24 hours, cells were washed with PBS (Sigma-Aldrich, U.K.) and re-incubated in 100μl of DMEM without FBS with glucose concentrations of 2mM, 5mM, and 25mM. After a 24-hour incubation, 10% Alamar Blue reagent was added to each well. Absorbance at 570 and 600 nm was measured with a microplate reader (Thermo LabSystems, USA), and Alamar Blue reduction was calculated following the manufacturer’s instructions. Each experiment was replicated three times with quadruplicates.

### Colony formation assay

MDA-MB-231 and MCF-7 cells, along with their resistant counterparts, underwent 24-hour exposure to hypoglycemia, normal glucose, and hyperglycemia. One million cells were initially seeded in 100 mm dishes with 25mM DMEM + FBS, followed by incubation. Post-incubation, cells were treated with 2mM, 5mM, and 25mM DMEM without FBS for 24 hours. After trypsinization, 500 cells/well were seeded in six-well plates and cultured for 14 days in complete growth media. Colonies were then stained with 25% methanol and 0.5% crystal violet, washed, and manually counted. The experiment was repeated three times.

### Analysis of apoptosis

The annexin V-FITC Apoptosis Detection Kit (BioVision, USA) was used to detect and quantify apoptosis by flow cytometry according to the manufacturer’s instructions.

Annexin V-FITC binding was analyzed by flow cytometry (FACS Aria III, .D.BD Biosciences, USA) using FITC signal detector and propidium iodide (P.I.) staining by the phycoerythrin emission signal detector. The data were analyzed using BD FACSDiva™ software.

### Invasion assay

Cell invasion was assessed with the CultreCoat 96 Well Medium BME assay (Trevigen, USA) using 25,000 cells/insert. Fluorescence was measured at 485 nm excitation and 520 nm emission via an Epoch Microplate Spectrophotometer (BioTek, USA) with Gen5 software version 2.07, following the manufacturer’s instructions.

### Quantitative PCR (qPCR)

MDA-MB-231, MCF7, and resistant cell RNA, extracted using TRI reagent, underwent cDNA synthesis with a high-capacity kit. Real-time PCR mixtures included Fast SYBR super mix, 15ng cDNA, and 100uM Tp53 primers. F.P.: TCTGTCCCTTCCCAGAAAACC RP: CAAGAAGCCCAGACGGAAAC. The 7500 Fast Real-time PCR system (Applied Biosystems, USA) was employed for gene expression quantification and analysis using the 2-ΔΔCt method for relative expression values.

#### Ethics declarations

There is no need to provide ethical approval for this study since this research was based on the use of cell lines only.

### Statistical analysis

Statistical analysis was performed on the means of at least three independent repeated experiments. Statistically significant differences were determined by the one-way ANOVA test P value less than 0.05 is considered significant for all tests.

## Supporting information

S1 Fig Hyperglycemia shields cells from Paclitaxel. Panel a (MDA-mB-231 control and resistant variants) and panel b (MCF7 control and resistant variants) show quantification of the average percentage of the cell death events after exposing the control and resistant cells to 50 mM of Paclitaxel for 24 hours without FBS under the three glucose levels (25 mM, 5 mM, and 2 mM). The experiment was repeated three independent times.
(TIF)

S1 Table List of primary antibodies used for the western blots. (DOCX)

S1 File Tp53 total graphs. (PDF)

S2 File Averaged invasion graphs. (PDF)

S1 Raw images (PDF)
