# Supplementary figures and images for: Glycemic load impacts the response of acquired resistance in breast cancer cells to chemotherapeutic drugs in vitro
Source: PLoS One. 2024 Nov 22;19(11):e0311345. doi: 10.1371/journal.pone.0311345 (PMC11584130; doi:10.1371/journal.pone.0311345)

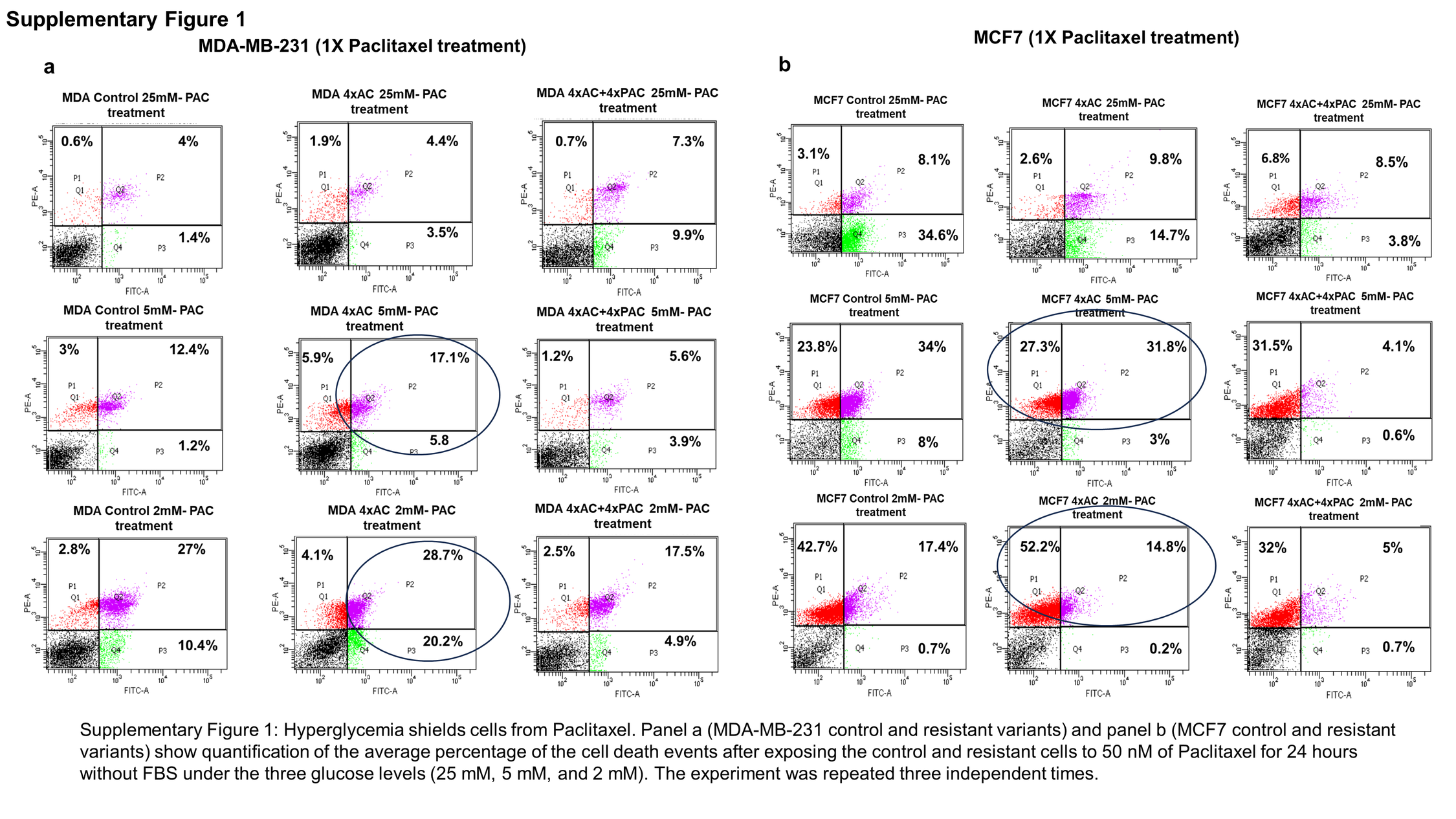

Supplement: S1 Fig — Panel a (MDA-mB-231 control and resistant variants) and panel b (MCF7 control and resistant variants) show quantification of the average percentage of the cell death events after exposing the control and resistant cells to 50 mM of Paclitaxel for 24 hours without FBS under the three glucose levels (25 mM, 5 mM, and 2 mM). The experiment was repeated three independent times. (TIF) [file pone.0311345.s001.tif]
