# Supplementary material for: Glycemic load impacts the response of acquired resistance in breast cancer cells to chemotherapeutic drugs in vitro
Source: PLoS One. 2024 Nov 22;19(11):e0311345. doi: 10.1371/journal.pone.0311345 (PMC11584130; doi:10.1371/journal.pone.0311345)
Supplement: S1 Table — (DOCX) [file pone.0311345.s002.docx]

Supplementary table 1: List of primary antibodies used for the western blots.

| **Antibody** | **Cat. #** | **Manufacturer** |
| --- | --- | --- |
| GAPDH (glyceraldehyde-3-phosphate dehydrogenase) | Rabbit mAb 5174 | Cell signalling |
| NRP-1 (Neuropilin-1) | Rabbit mAb ab81321 | Abcam |
| p-NF-ĸB _P56_ (Pospho-Nuclear factor kappa B) | Rabbit mAb #3033 | Cell signalling |
| NF-ĸB _P56_ (Nuclear factor kappa B) | Rabbit mAb #8242 | Cell signalling |
| p-Akt (S473) | Rabbit mAb #4060 | Cell signalling |
| Akt | Rabbit mAb #4691 | Cell signalling |
| Phospho-GSK-3β (Ser9)  GSK-3β (D5C5Z) XP® Rabbit mAb #12456  IGF1R  P27 | Rabbit mAb #9336  Rabbit mAb #12456  Rabbit mAb #9750S  Rabbit mAb #3686 | Cell signalling  Cell signalling  Cell signalling  Cell signalling  Cell signalling |
|  |  |  |
|  |  |  |
|  |  |  |
|  |  |  |
|  |  |  |
