# Supplementary material for: Glycemic load impacts the response of acquired resistance in breast cancer cells to chemotherapeutic drugs in vitro
Source: PLoS One. 2024 Nov 22;19(11):e0311345. doi: 10.1371/journal.pone.0311345 (PMC11584130; doi:10.1371/journal.pone.0311345)
Supplement: S1 File — (PDF) [file pone.0311345.s003.pdf]

- **TP53 expression in MDA-MB-231 cells**

MDA-MB-231	Glucose Concentration (mM)	q-PCR #1	q-PCR #2	Avg	stdev
MDA Control	2	1	1	1	0
	5	0.8875345	1.1167391	1.00213683	0.16207214
	25	0.6540627	0.6703175	0.66219006	0.01149388
MDA-4xAC	2				
	5	0.6983801	0.7203918	0.70938591	0.01556462
	25	0.7155621	0.5428651	0.62921363	0.12211523
MDA-4xPAC	2	1.6251437	0.9708596	1.29800161	0.46264872
	5	1.2359515	1.3927311	1.31434128	0.11085993
	25	0.3792189	0.3709155	0.3750672	0.00587141

- **TP53 expression in MCF-7 cells**

		q-PCR #1	q-PCR #2	Avg	stdev
MCF7 Control	25mM	1.1967224	0.9854921	1.09110723	0.14936239
	5mM	**2.9833059**	**0.7478229**	1.86556442	1.58072519
	2mM	1	1		
MCF7 4xAC	25mM	1.0096965	0.6014838	0.80559015	0.28865
	5mM	0.9378746	0.3112638	0.62456919	0.4430807
	2mM	2.1068541	0.3871208	1.24698743	1.21603511
MCF7 4xAC+4xPAC	25mM	0.0862624	0.0247971	0.05552978	0.04346253
	5mM	0.0045561	0.0207227	0.0126394	0.01143148
	2mM	0.0263218	0.0173384	0.02183013	0.00635221

		Avg	SEM
MCF7 Control	25mM	1.09110723	0.10561516
	5mM	1.86556442	1.1177415
	2mM		
MCF7 4xAC	25mM	0.80559015	0.20410637
	5mM	0.62456919	0.31330537
	2mM	1.24698743	0.85986668
MCF7	25mM	0.05552978	0.03073265
	5mM	0.0126394	0.00808328
	2mM	0.02183013	0.00449169

MDA-MB-231	Glucose Concentration (mM)	Avg	stdev	SEM
MDA Control	25mM	0.66219006	0.01149388	0.0081274

	5mM	1.00213683	0.16207214	0.11460231
	2mM	1	0	0
MDA-4xAC	25mM	0.62921363	0.12211523	0.08634851
	5mM	0.70938591	0.01556462	0.01100585
	2mM			0
MDA-4xAC+4xPAC	25mM	0.3750672	0.00587141	0.00415172
	5mM	1.31434128	0.11085993	0.0783898
	2mM	1.29800161	0.46264872	0.32714204

- **TP53 expression in MDA-MB-231 cells**

MDA-MB-231	Glucose Concentration (mM)	q-PCR #1	q-PCR #2
MDA Control	2	1	1
	5	0.8875345	1.1167391
	25	0.6540627	0.6703175
MDA-4xAC	2		
	5	0.6983801	0.7203918
	25	0.7155621	0.5428651
MDA-4xPAC	2	1.6251437	0.9708596
	5	1.2359515	1.3927311
	25	0.3792189	0.3709155

MDA Control

MDA-4xAC

MDA 4xAC+4

- **TP53 expression in MCF-7 cells**

		q-PCR #1	q-PCR #2
MCF7 Control	25mM	1.1967224	0.9854921
	5mM	**2.9833059**	**0.7478229**
	2mM	1	1
MCF7 4xAC	25mM	1.0096965	0.6014838
	5mM	0.9378746	0.3112638

MCF7 Control

MCF7 4xAC

	2mM	2.1068541	0.3871208
MCF7 4xAC+4xPAC	25mM	0.0862624	0.0247971
	5mM	0.0045561	0.0207227
	2mM	0.0263218	0.0173384

MCF7 4xAC+4

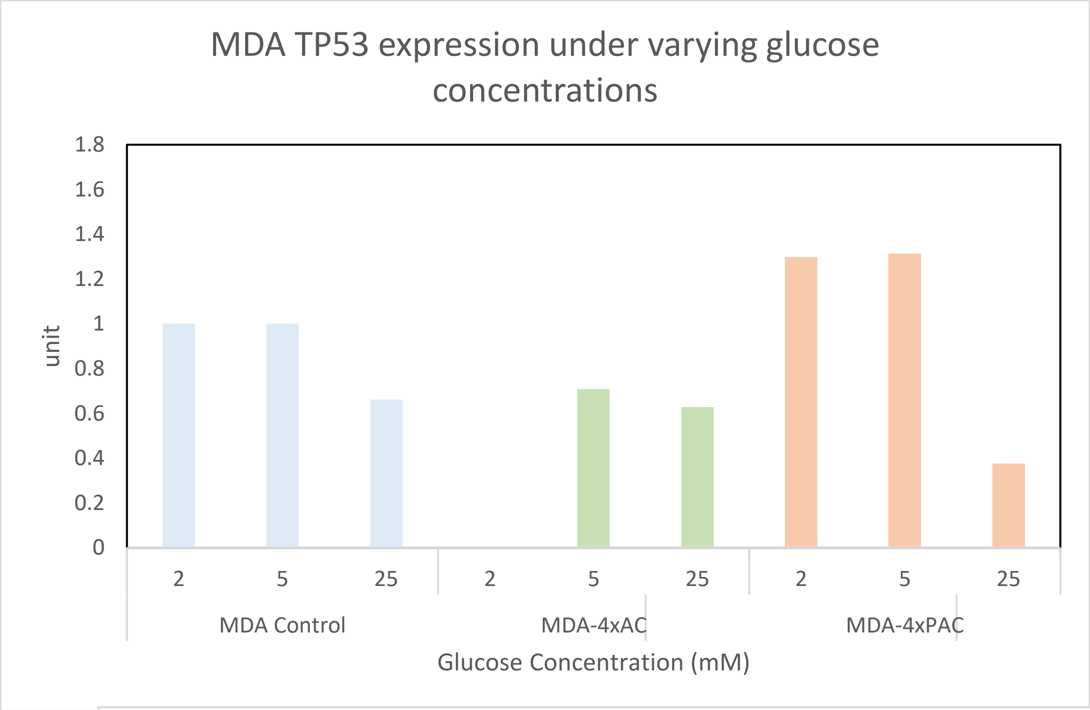

SEM

0.10561516

1.1177415

0.20410637

0.31330537

0.85986668

0.03073265

0.00808328

0.00449169

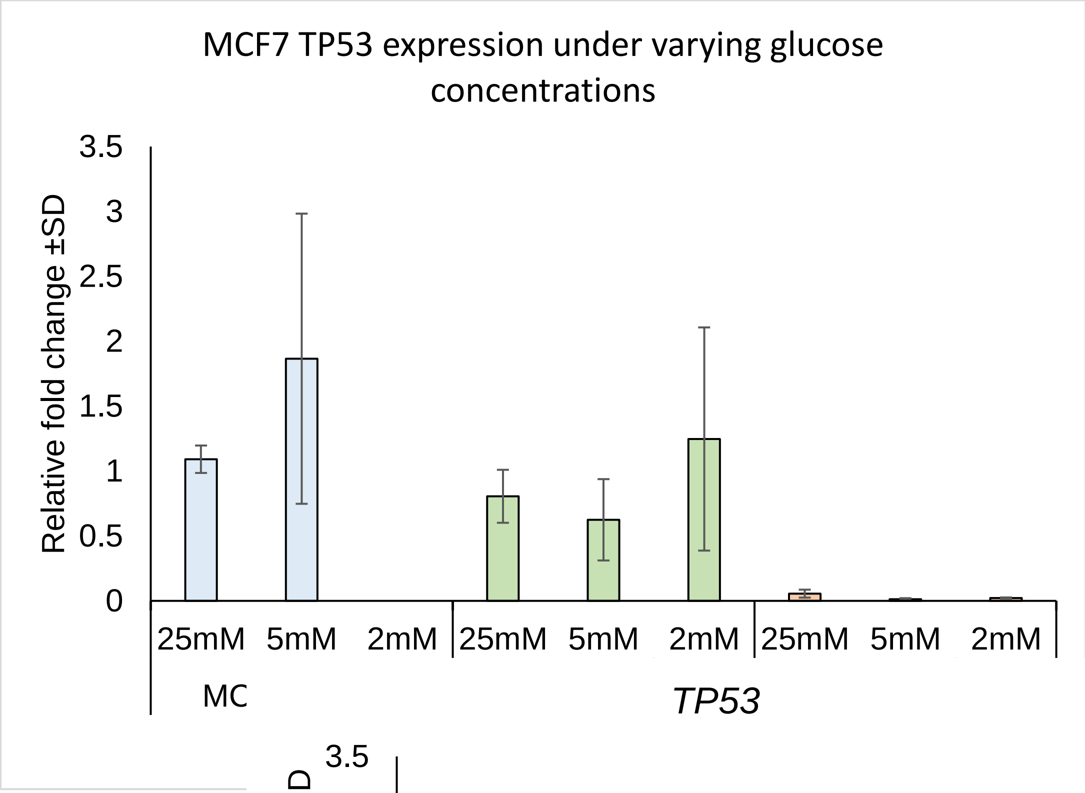

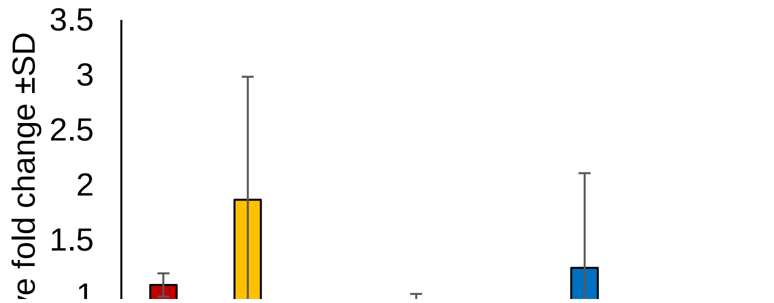

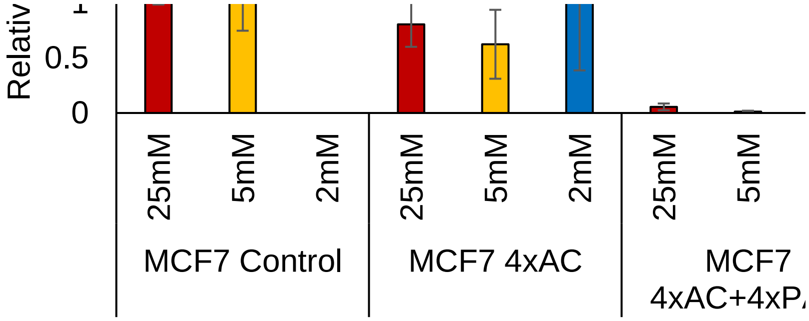

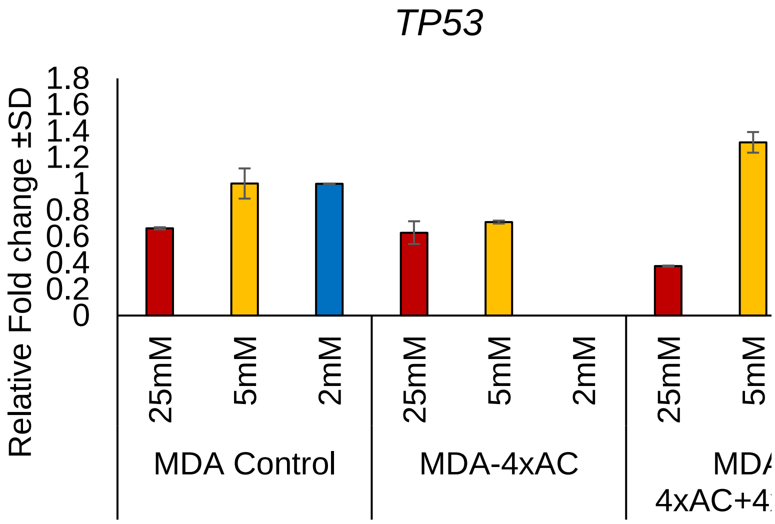

	q-PCR #1	q-PCR #2
2mM	1	1
5mM	0.88753452	1.11673914
25mM	0.65406266	0.67031746
2mM		
5mM	0.69838006	0.72039175
25mM	0.71556214	0.54286512
2mM	1.62514365	0.97085956
5mM	1.23595147	1.39273108
25mM	0.37921891	0.37091548

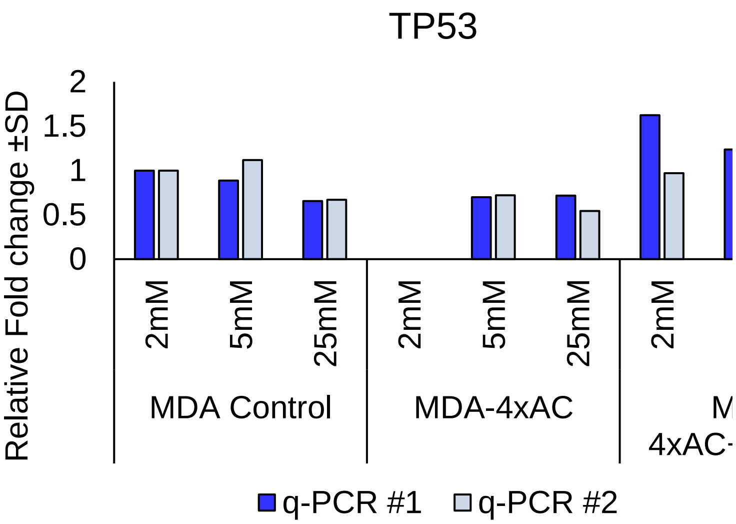

	q-PCR #1	q-PCR #2
25mM	1.19672239	0.98549207
5mM	2.98330592	0.74782292
2mM	1	1
25mM	1.00969652	0.60148378
5mM	0.93787456	0.31126382

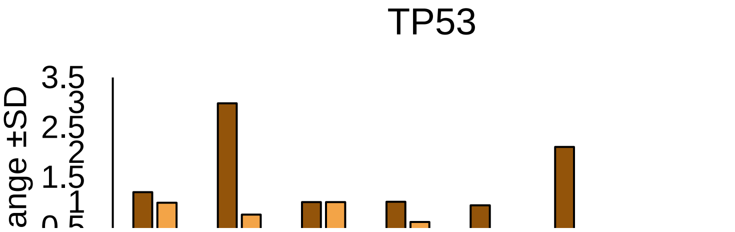

2mM	2.1068541	0.38712075
25mM	0.08626243	0.02479713
5mM	0.00455612	0.02072268
2mM	0.02632182	0.01733844

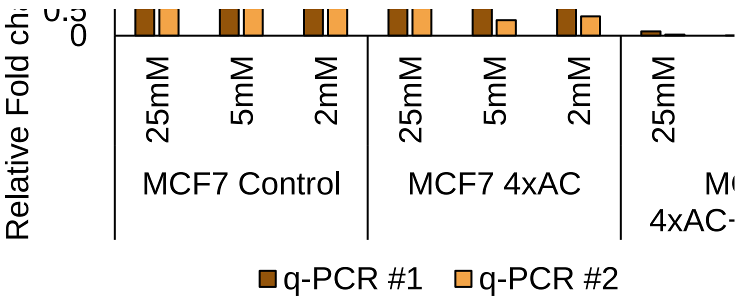

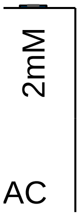
2mM
AC

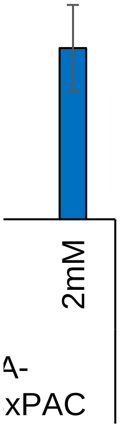
2mM
A-
xPAC

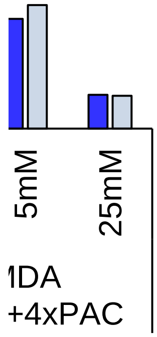
5mM
25mM
MDA
+4xPAC

5mM 2mM

CF7
+4xPAC
