# Supplementary material for: Glycemic load impacts the response of acquired resistance in breast cancer cells to chemotherapeutic drugs in vitro
Source: PLoS One. 2024 Nov 22;19(11):e0311345. doi: 10.1371/journal.pone.0311345 (PMC11584130; doi:10.1371/journal.pone.0311345)

MCF7

	Avg 1	Avg 2	Avg 3	STDEV
MCF7-2	9	10	9.5	0.5
MCF7-5	7	8.66666667	7.83333333	0.83333333
MCF7-25	8.66666667	6.66666667	7.66666667	1
MCF7-4XAC-2	4.3333333	6.33333333	5.33333332	1.00000002
MCF7-4XAC-5	3	6.33333333	4.66666667	1.66666667
MCF7-4XAC-25	2.3333333	6.33333333	4.33333332	2.00000002
MCF7-4XAC+PAC-2	5.33333	6.33333333	5.83333167	0.50000167
MCF7-4XAC+PAC-5	6	8	7	1
MCF7-4XAC+PAC-25	2	6.33333333	4.16666667	2.16666667
	2mM	5.83333167	0.70710914	3.62478671
	5mM	7	1.41421356	3.94974747
	25mM	4.16666667	3.06412939	0.77961159

MCF7 control
MCF7 4xAC
MCF7 4xAC+4xPAC

MDA-MB-231

	Glucose Con	Avg	stdev	SEM
	25mM	46	6.55743852	3.7859389
	5mM	36.3333333	5.03322296	2.90593263
MDA control	2mM	23	6.55743852	3.7859389
	25mM	34	4	2.30940108
	5mM	36.6666667	8.6216781	4.97772817
MDA-4xAC	2mM	37.6666667	5.03322296	2.90593263
	25mM	73.6666667	1.52752523	0.8819171
	5mM	47.3333333	4.72581563	2.72845092
MDA-4xAC+4xPAC	2mM	43.3333333	5.13160144	2.96273147

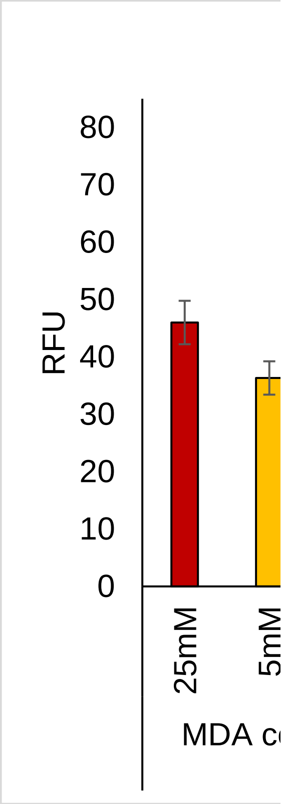

Glucose Conc	avg	stdev	SEM
25mM	7.66666667	1	
5mM	7.83333333	0.83333333	
2mM	9.5	0.5	
25mM	4.33333332	2.00000002	
5mM	4.66666667	1.66666667	
2mM	5.33333332	1.00000002	
25mM	4.16666667	2.16666667	
5mM	7	1	
2mM	5.83333167	0.50000167	

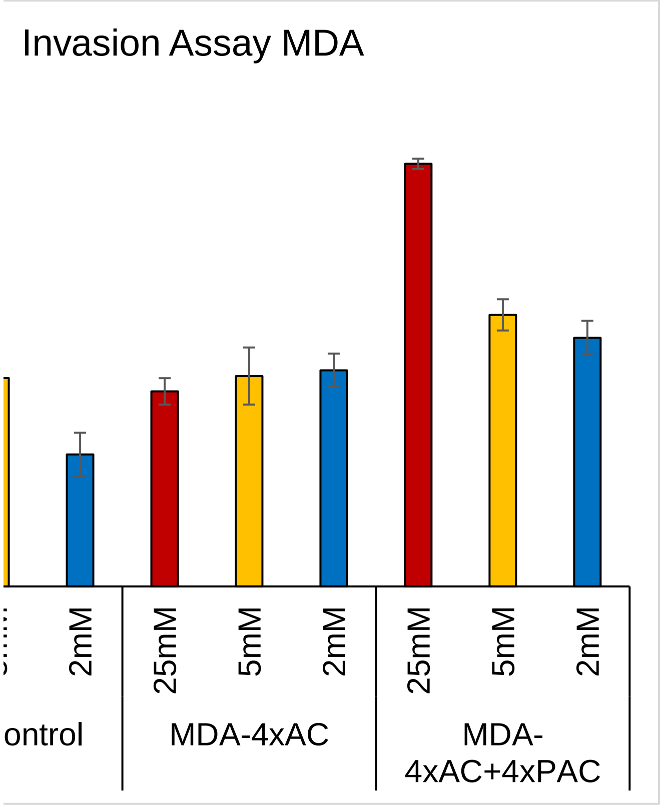

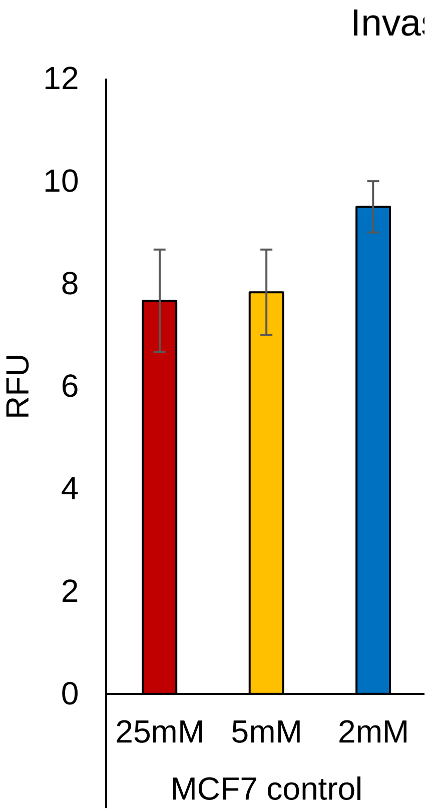

sion Assay MCF7

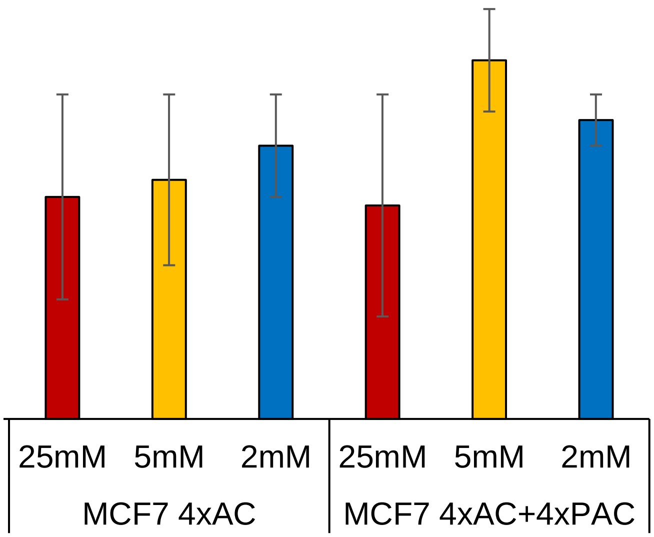

Supplement: S2 File — (PDF) [file pone.0311345.s004.pdf]
