# Supplementary material for: Glycemic load impacts the response of acquired resistance in breast cancer cells to chemotherapeutic drugs in vitro
Source: PLoS One. 2024 Nov 22;19(11):e0311345. doi: 10.1371/journal.pone.0311345 (PMC11584130; doi:10.1371/journal.pone.0311345)

Original blot as captured using the image lab software using the Chemidoc touch system BioRad In Figure 5 a

Figure 5 a

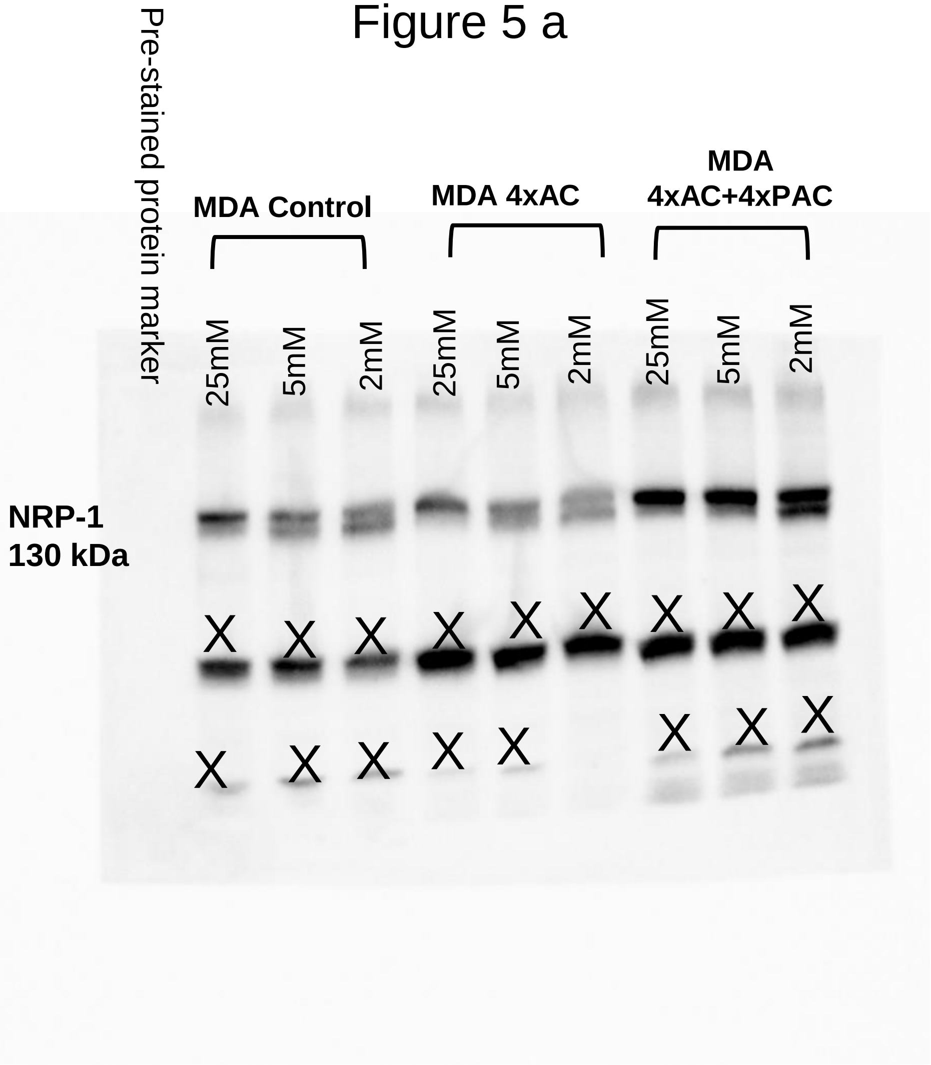

Original blot as captured using the image lab software using the Chemidoc touch system BioRad
In Figure 5 a

Figure 5 a

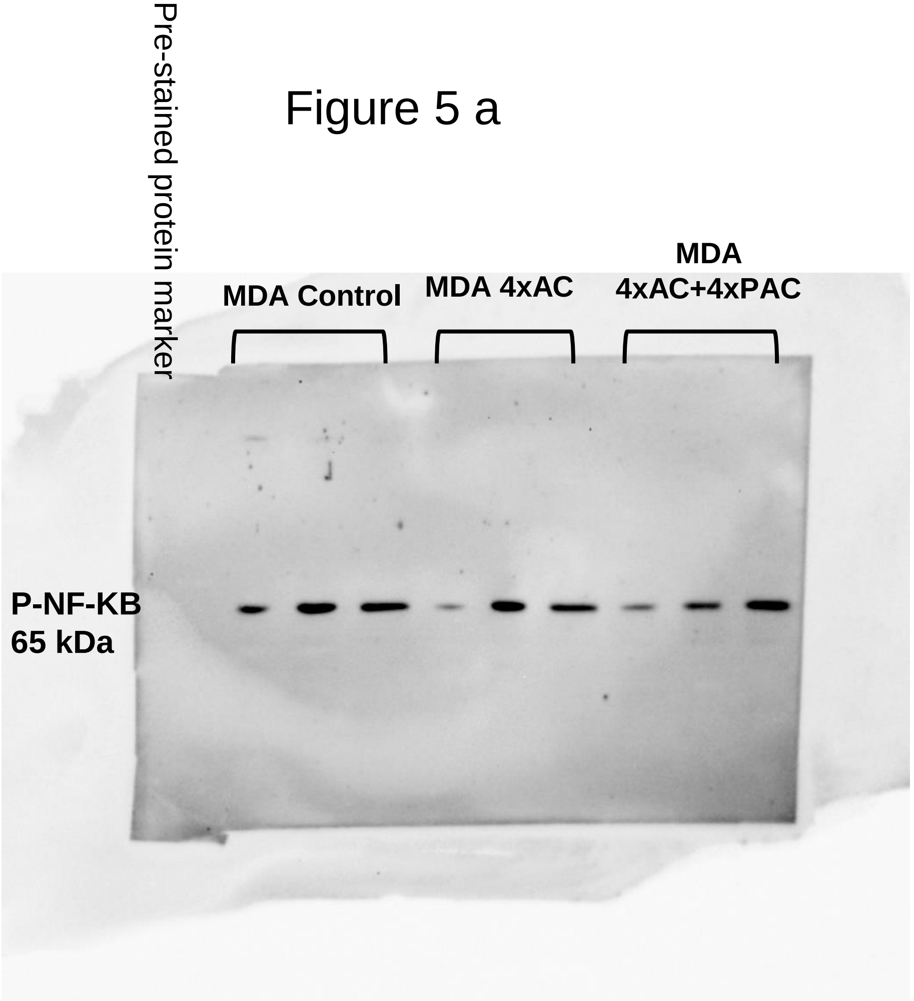

Original blot as captured using the image lab software using the Chemidoc touch system BioRad
In Figure 5 a

Figure 5 a

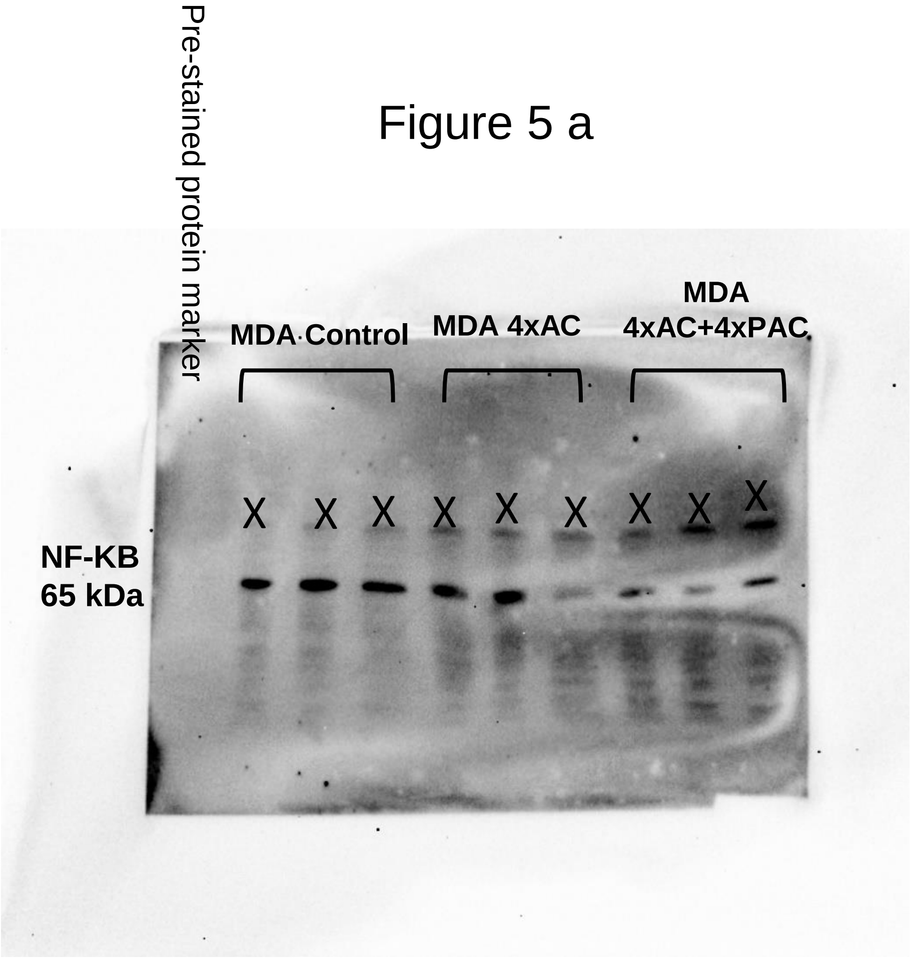

Original blot as captured using the image lab software using the Chemidoc touch system BioRad In Figure 5 a

Figure 5 a

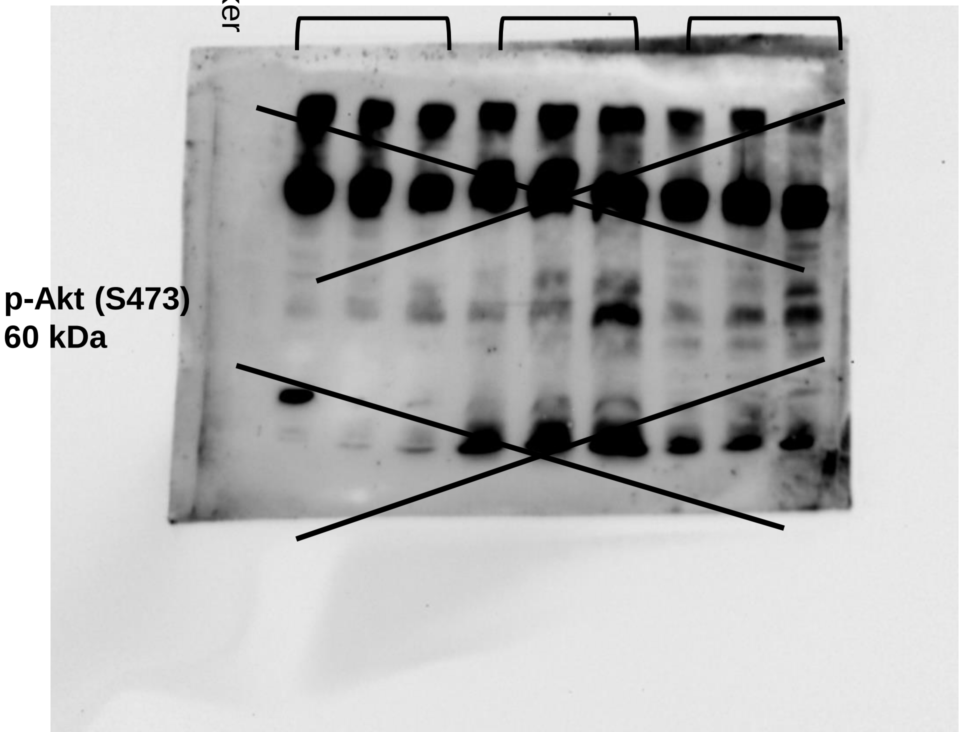

Original blot as captured using the image lab software using the Chemidoc touch system BioRad In Figure 5 a

Figure 5 a

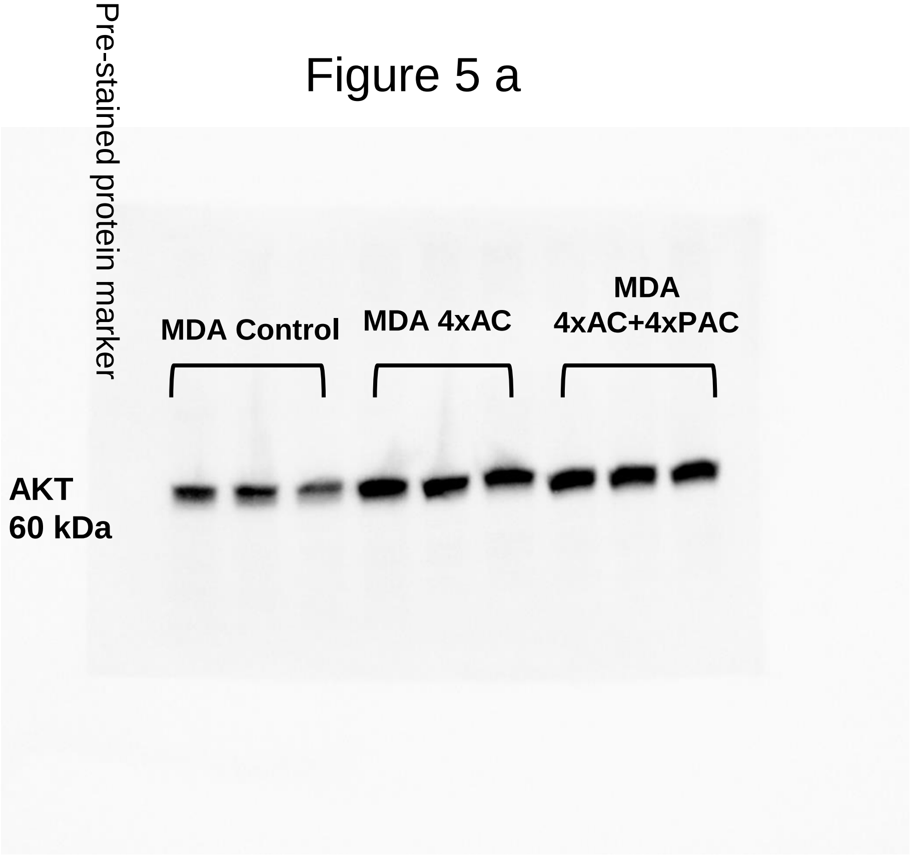

Original blot as captured using the image lab software using the Chemidoc touch system BioRad in Figure 5a

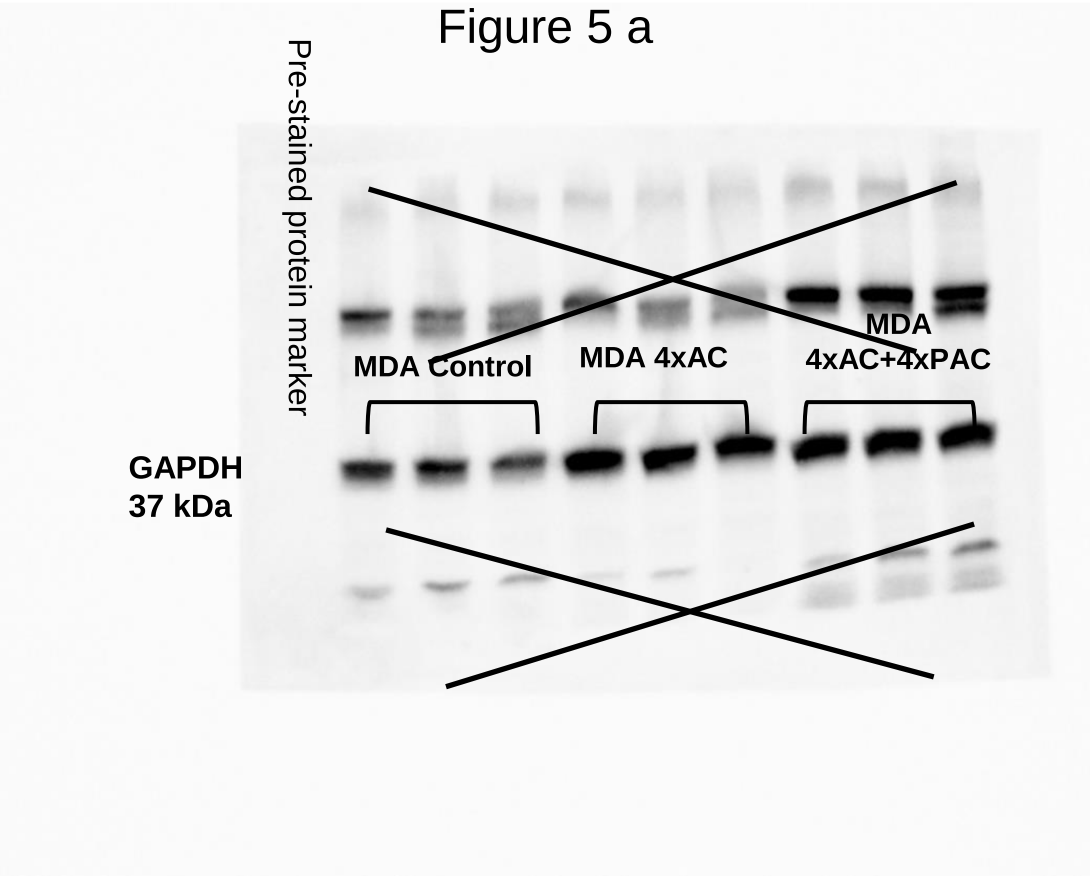

Original blot as captured using the image lab software using the Chemidoc touch system BioRad in Figure 5 b-

Figure 5 b

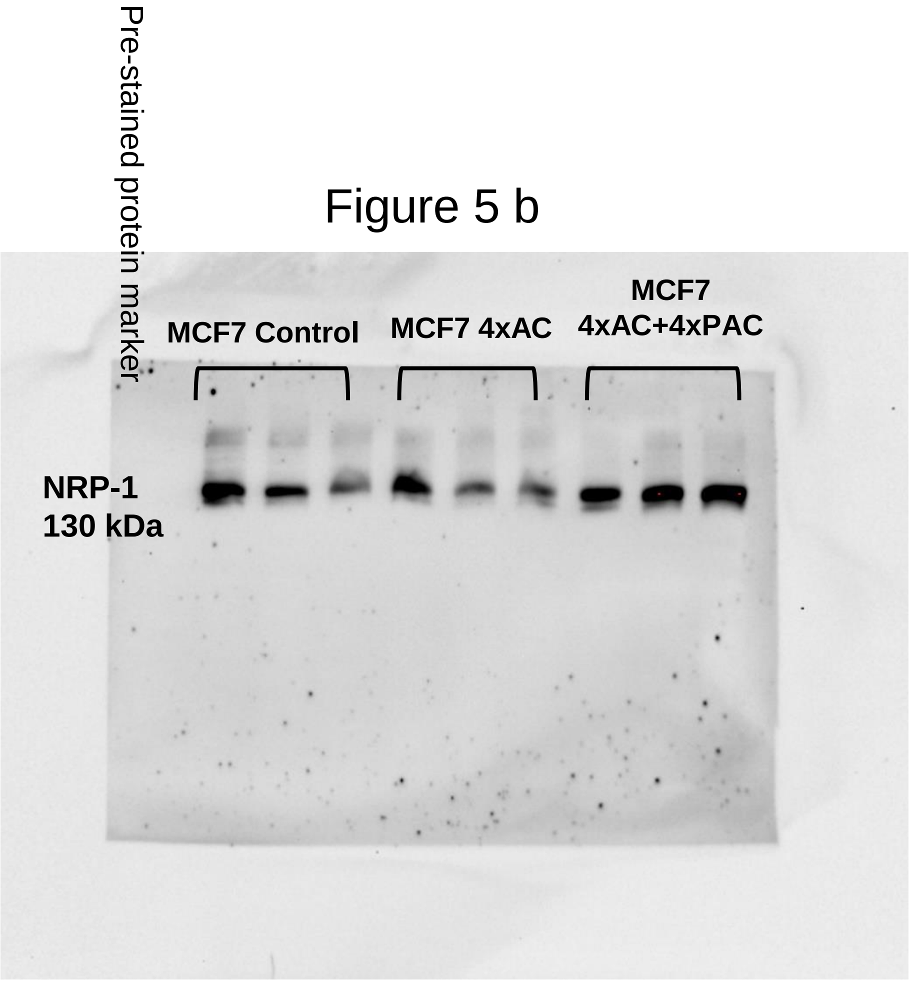

Original blot as captured using the image lab software using the Chemidoc touch system BioRad in Figure 5 b

Figure 5 b

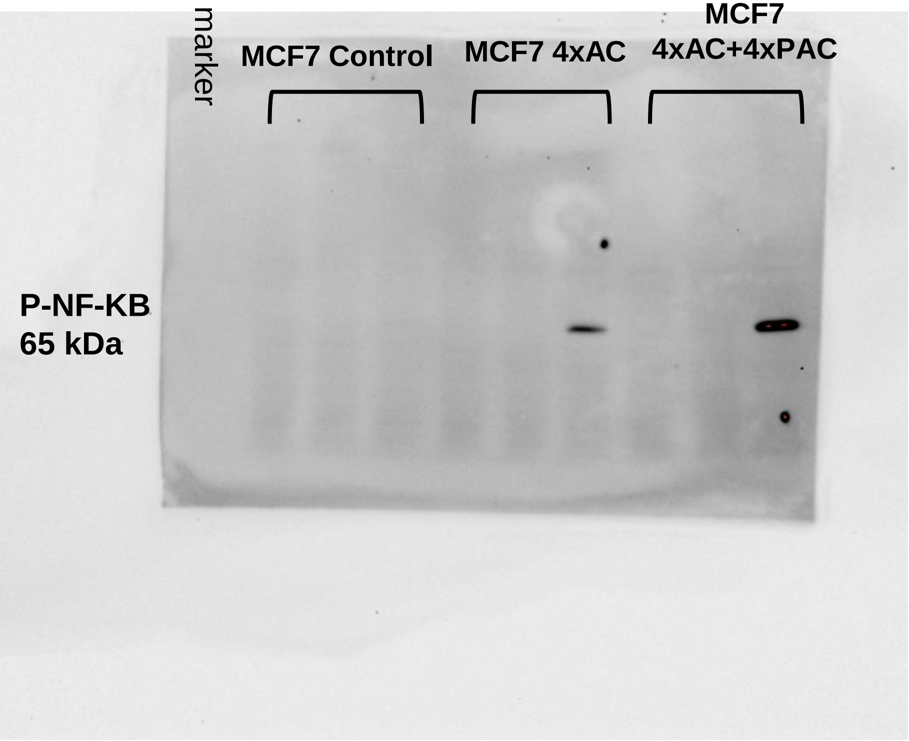

Original blot as captured using the image lab software using the Chemidoc touch system BioRad in Figure 5 b

Figure 5 b

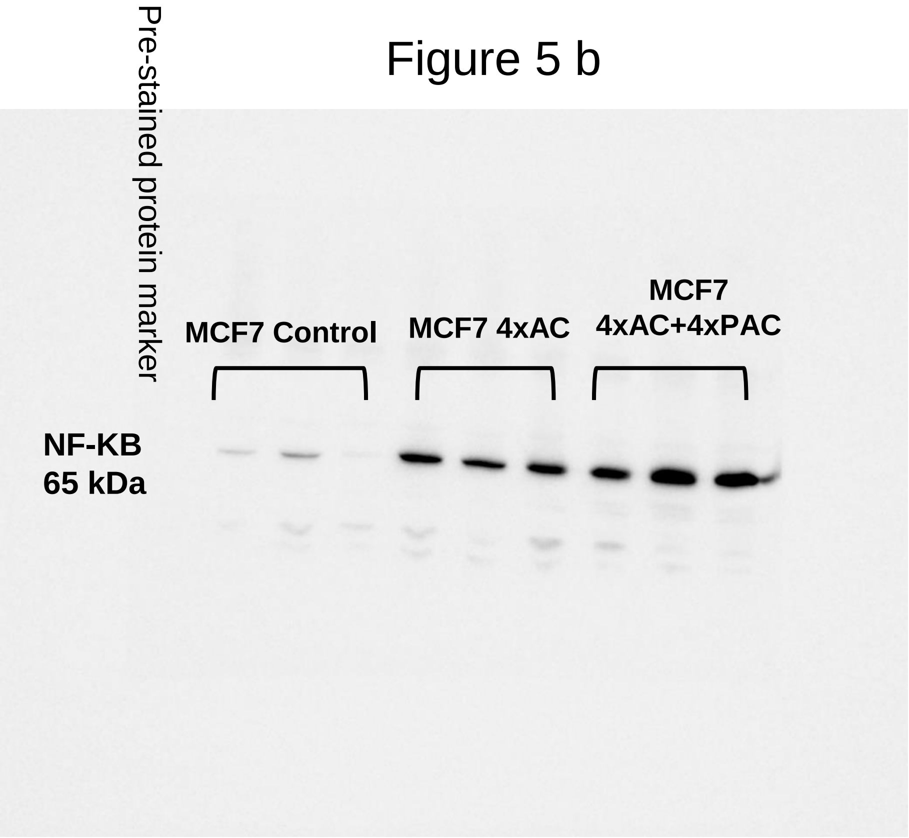

Original blot as captured using the image lab software using the Chemidoc touch system BioRad in Figure 5 b

Figure 5 B

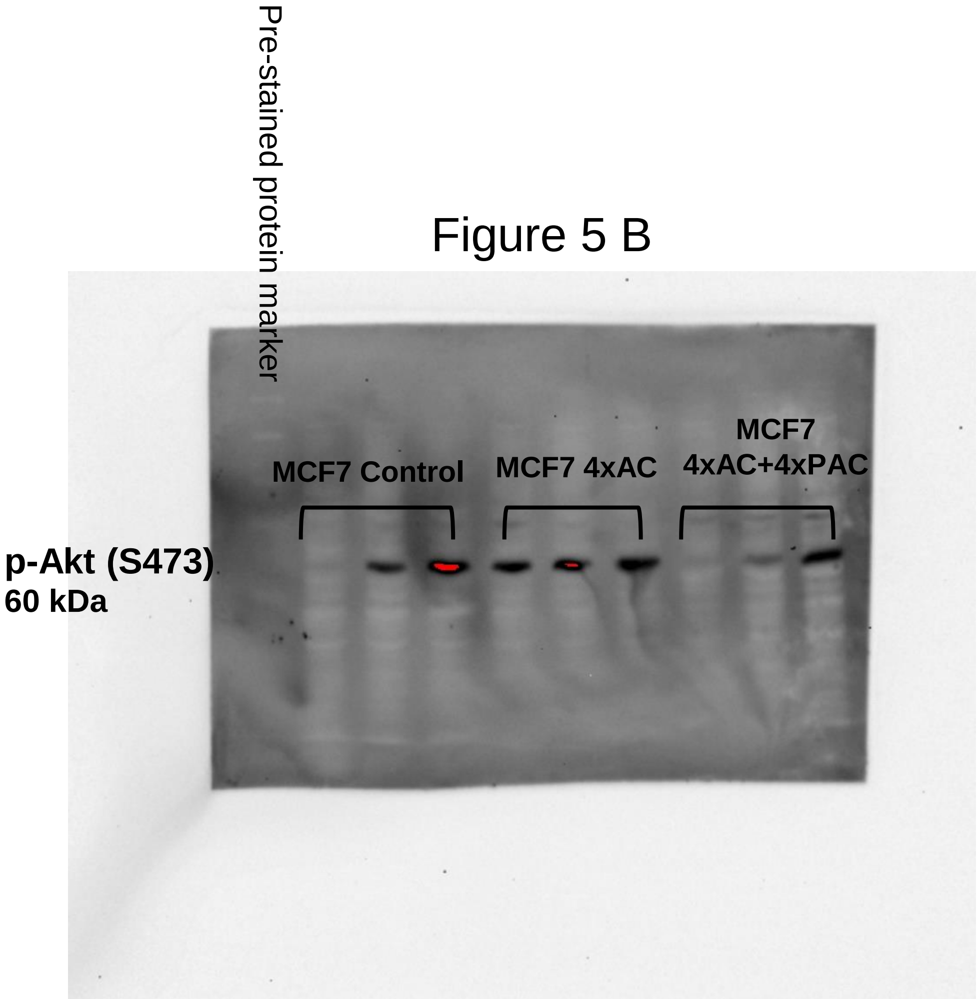

Original blot as captured using the image lab software using the Chemidoc touch system BioRad in Figure 5 b

Figure 5 b

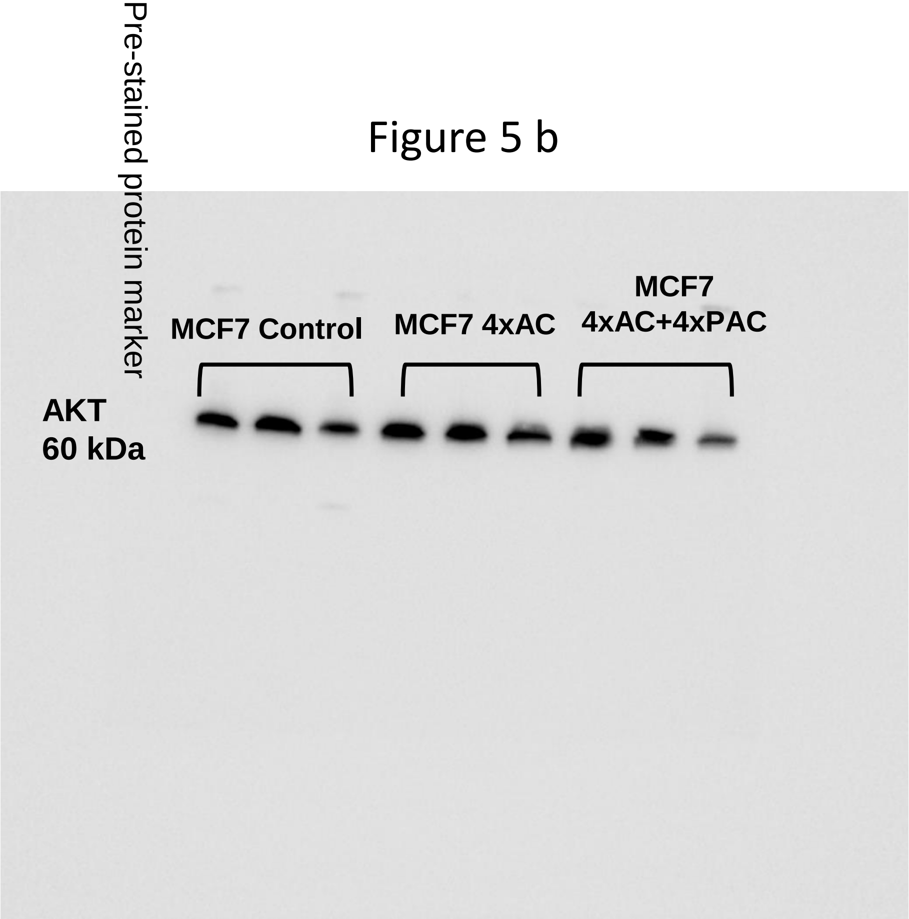

Figure 5 b

Original blot as captured using the image lab software using the Chemidoc touch system BioRad in Figure 5 b

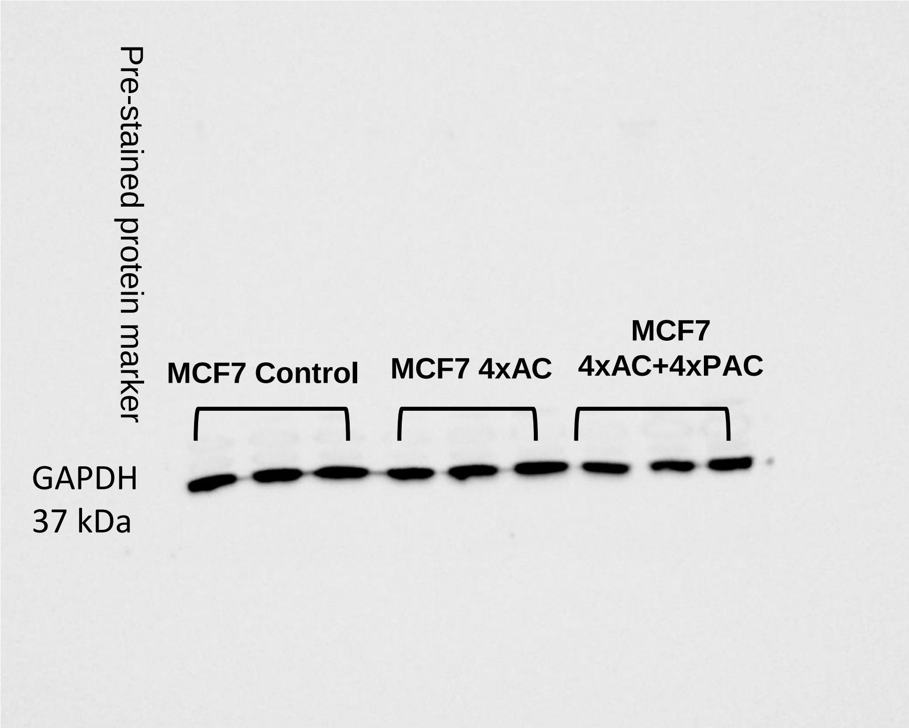

Figure 5 c

Original blot as captured using the image lab software using the Chemidoc touch system BioRad in Figure 5 c

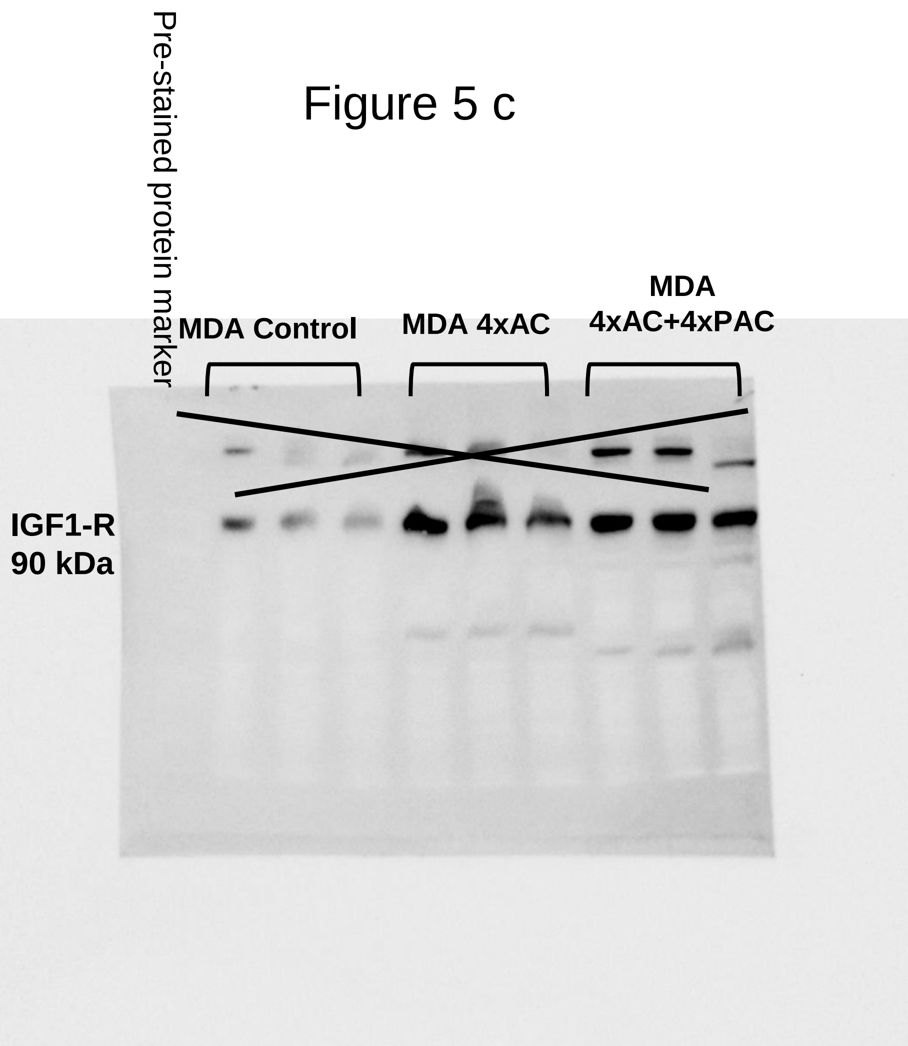

Figure 5 c

Original blot as captured using the image lab software using the Chemidoc touch system BioRad in Figure 5 c

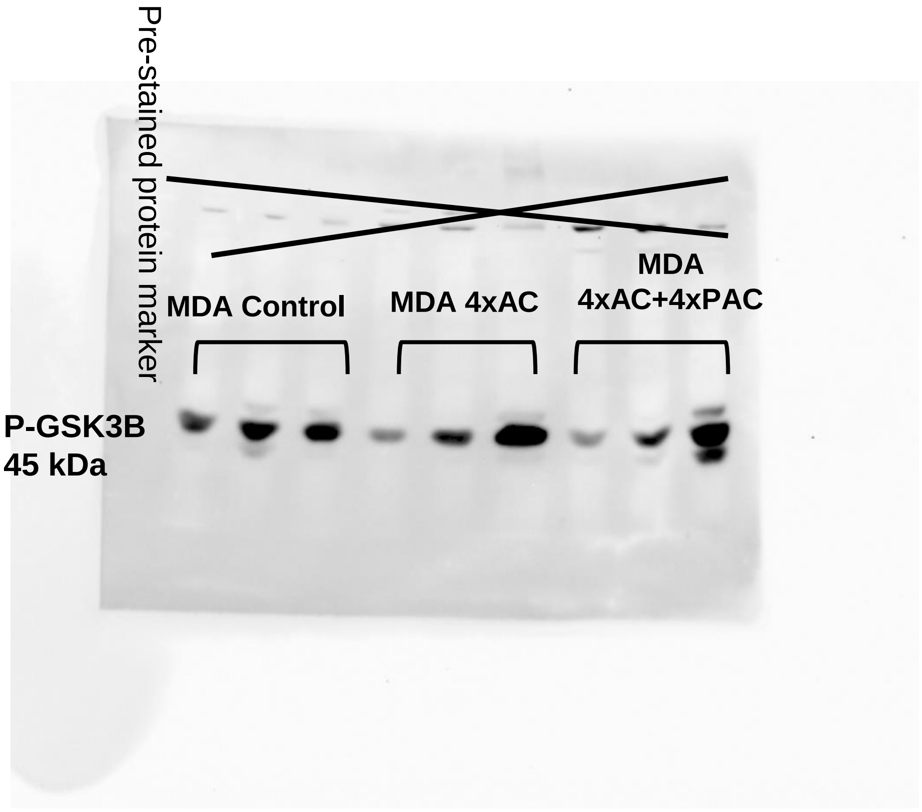

Figure 5 c

Original blot as captured using the image lab software using the Chemidoc touch system BioRad in Figure 5 c

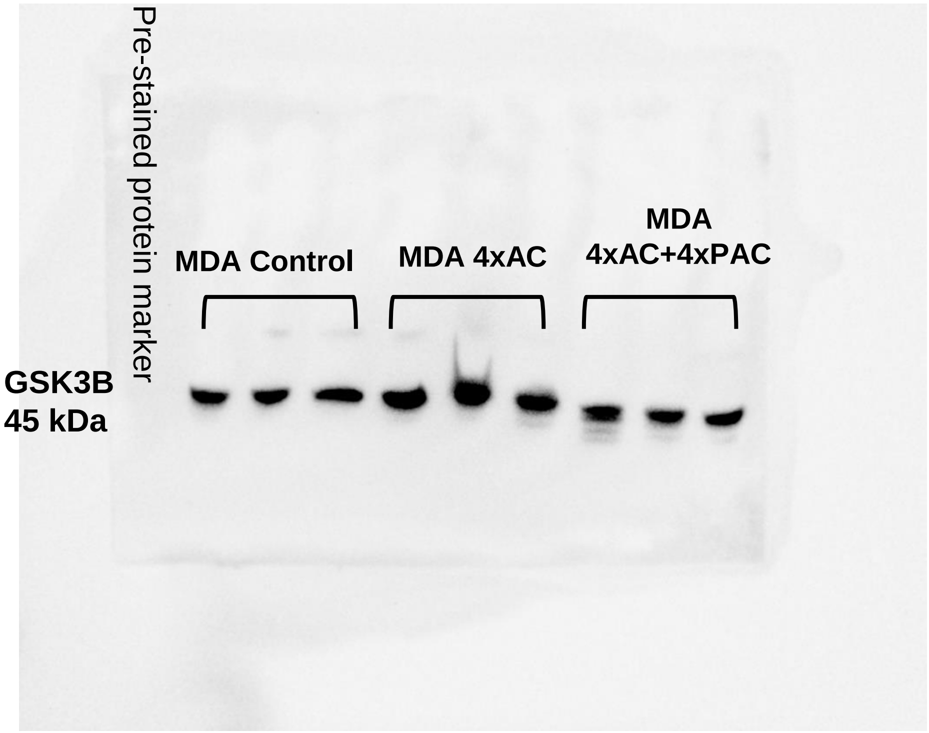

Original blot as captured using the image lab software using the Chemidoc touch system BioRad in Figure 5 c

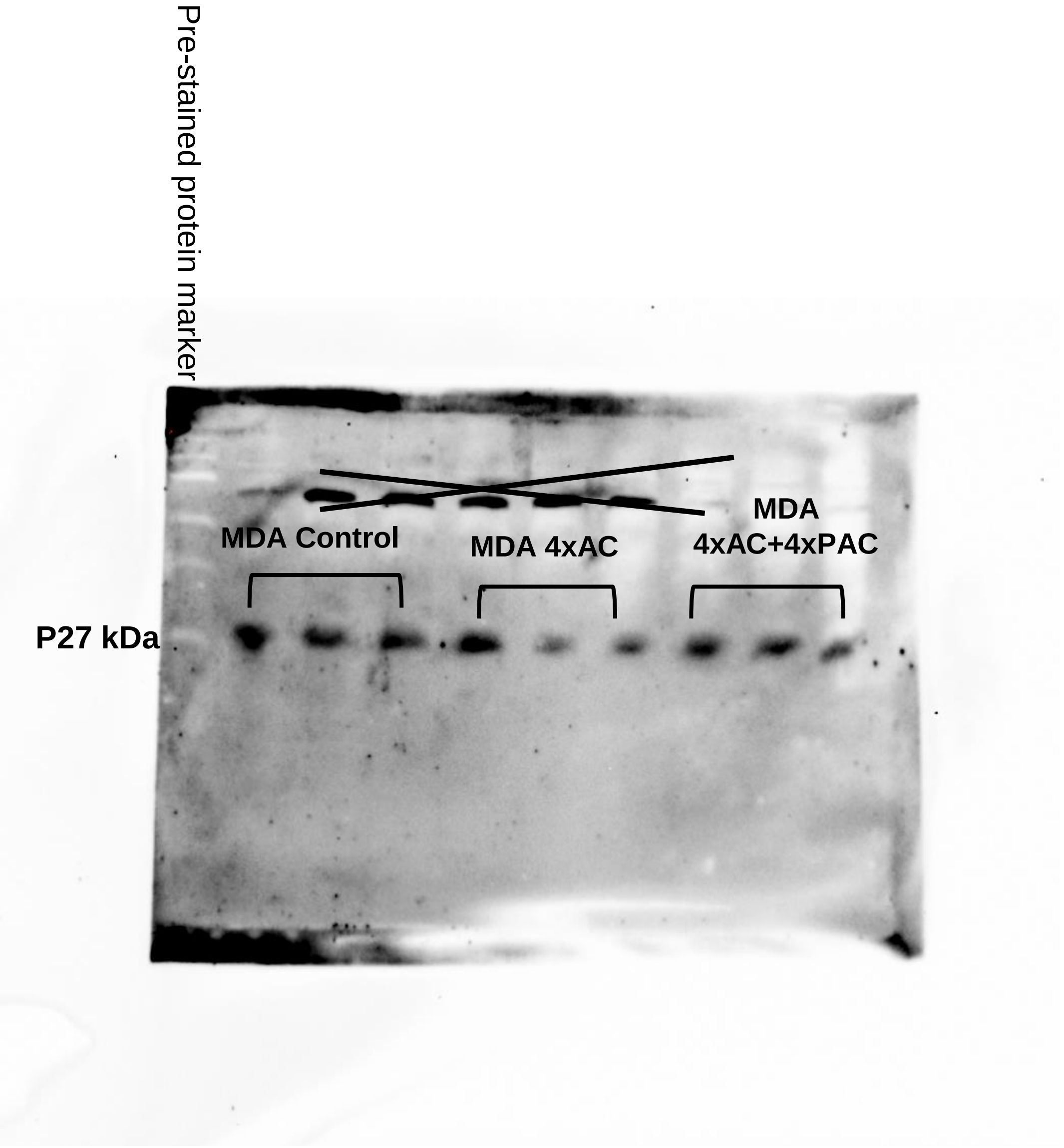

Figure 5 c

Original blot as captured using the image lab software using the Chemidoc touch system BioRad in Figure 5 c

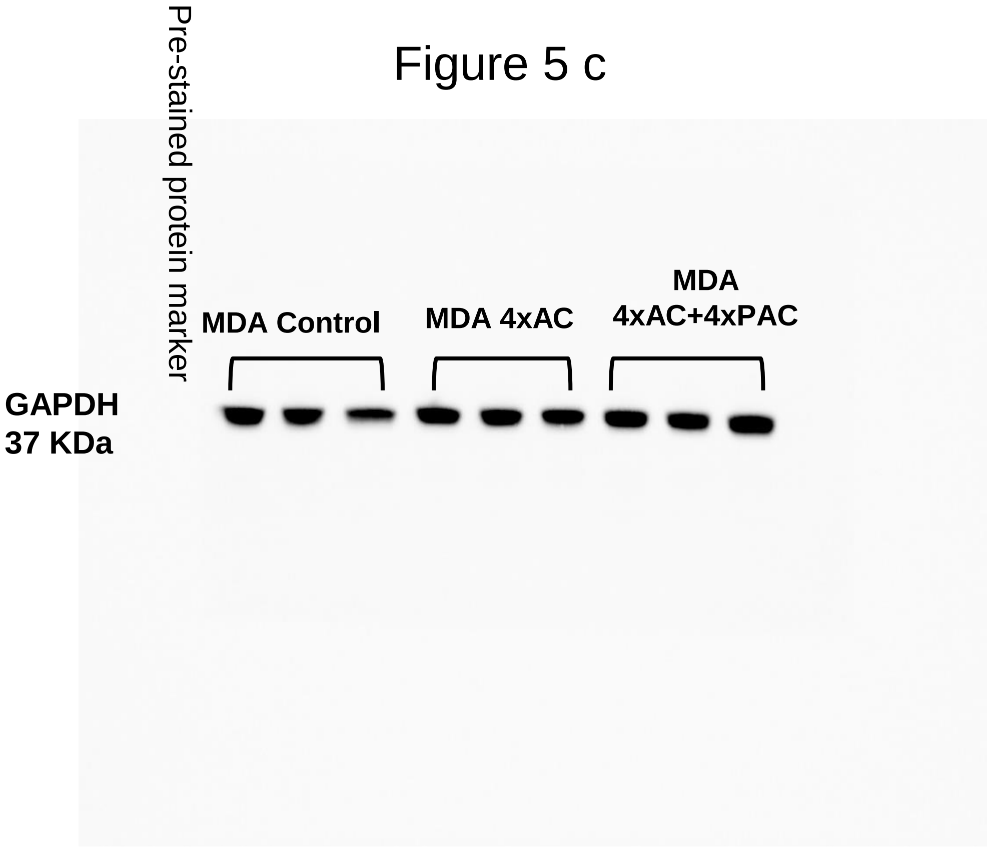

Original blot as captured using the image lab software using the Chemidoc touch system BioRad in Figure 5 d

Figure 5 d

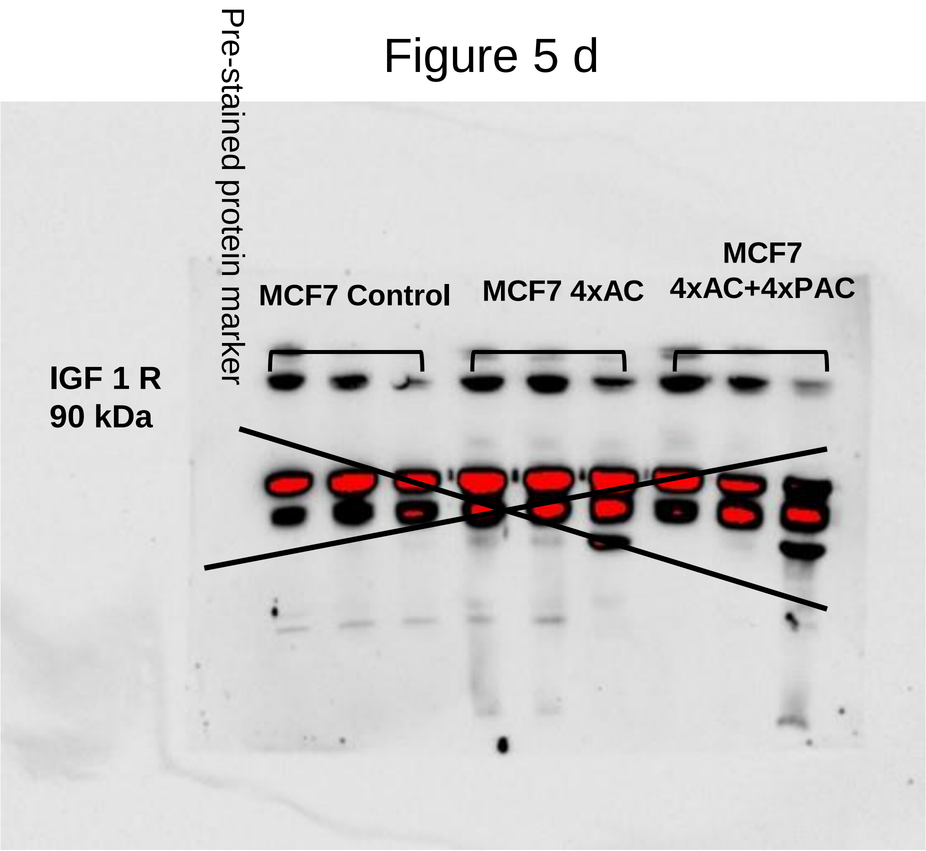

Original blot as captured using the image lab software using the Chemidoc touch system BioRad in Figure 5 d

Figure 5 d

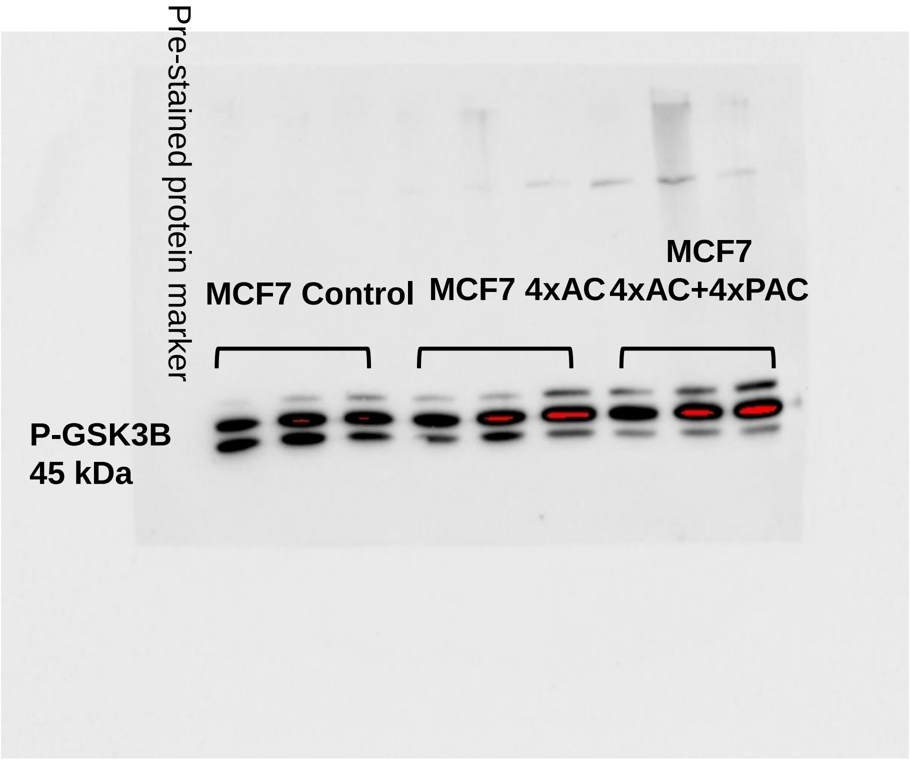

Figure 5 d

Original blot as captured using the image lab software using the Chemidoc touch system BioRad in Figure 5 d

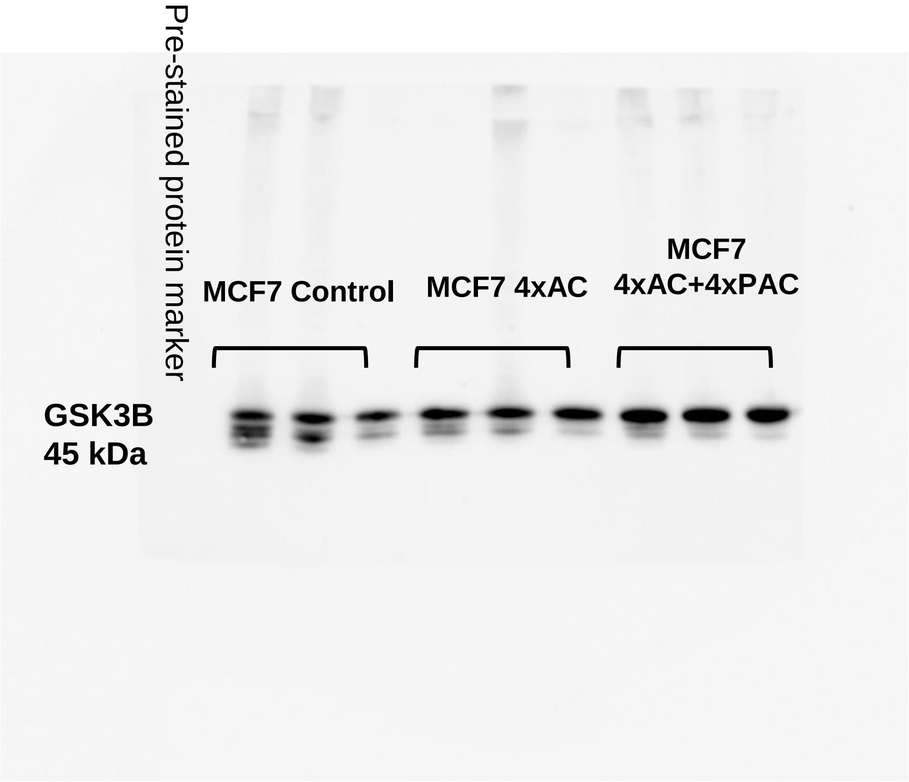

Original blot as captured using the image lab software using the Chemidoc touch system BioRad in Figure 5 d

Figure 5 d

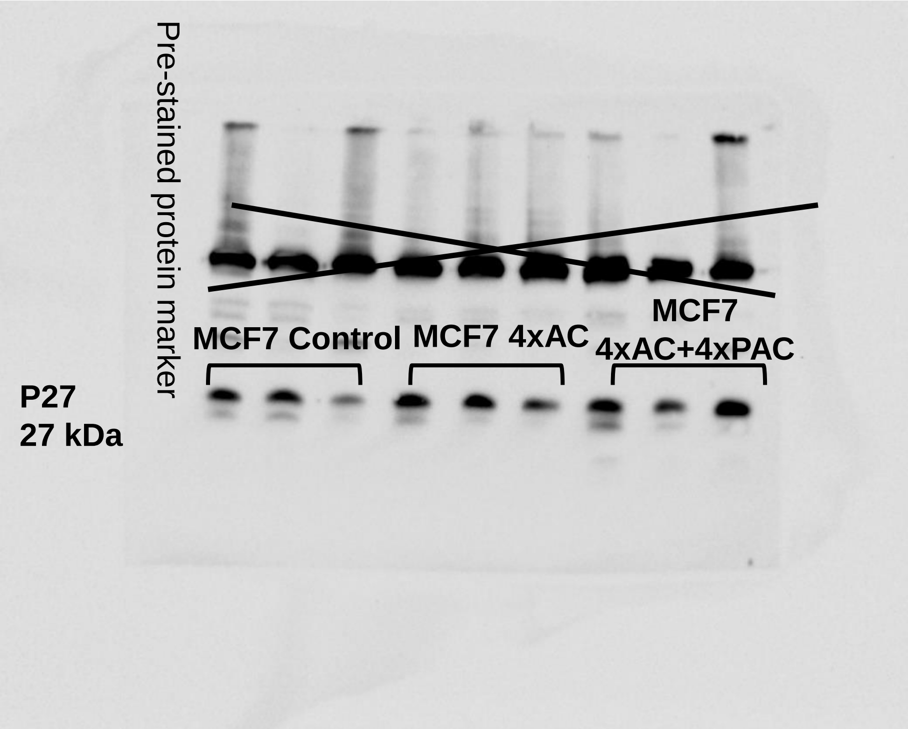

Figure 5 d

Original blot as captured using the image lab software using the Chemidoc touch system BioRad in Figure 5 d

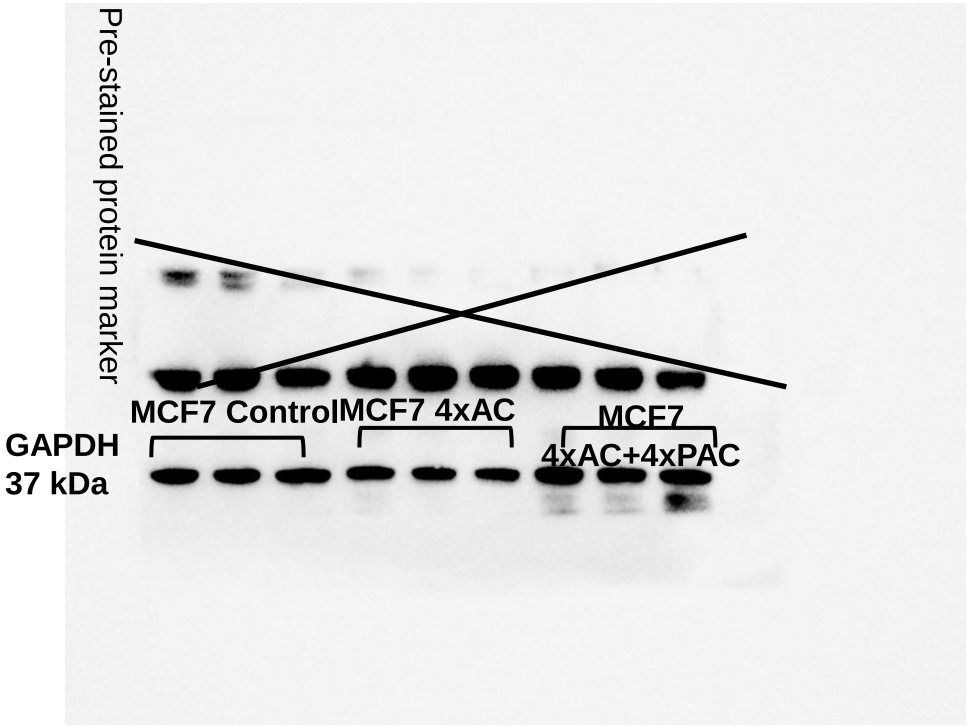

Supplement: S1 Raw images — (PDF) [file pone.0311345.s005.pdf]
